# Spermine inhibits PAMP-induced ROS and Ca^2+^ burst and reshapes the transcriptional landscape of PAMP-triggered immunity in Arabidopsis

**DOI:** 10.1093/jxb/erac411

**Published:** 2022-10-20

**Authors:** Chi Zhang, Kostadin E Atanasov, Rubén Alcázar

**Affiliations:** Department of Biology, Healthcare and Environment. Section of Plant Physiology, Faculty of Pharmacy and Food Sciences, Universitat de Barcelona, Av. Joan XXIII 27-31, 08028 Barcelona, Spain; Department of Biology, Healthcare and Environment. Section of Plant Physiology, Faculty of Pharmacy and Food Sciences, Universitat de Barcelona, Av. Joan XXIII 27-31, 08028 Barcelona, Spain; Department of Biology, Healthcare and Environment. Section of Plant Physiology, Faculty of Pharmacy and Food Sciences, Universitat de Barcelona, Av. Joan XXIII 27-31, 08028 Barcelona, Spain; Sichuan Agricultural University, China

**Keywords:** Defense, NADPH oxidase, pathogen-associated molecular pattern, polyamines, reactive oxygen species, putrescine, spermine

## Abstract

Polyamines are small polycationic amines whose levels increase during defense. Previous studies support the contribution of the polyamine spermine to defense responses. However, the potential contribution of spermine to pathogen-associated molecular pattern (PAMP)-triggered immunity (PTI) has not been completely established. Here, we compared the contribution of spermine and putrescine to early and late PTI responses in Arabidopsis. We found that putrescine and spermine have opposite effects on PAMP-elicited reactive oxygen species (ROS) production, with putrescine increasing and spermine lowering the flg22-stimulated ROS burst. Through genetic and pharmacological approaches, we found that the inhibitory effect of spermine on flg22-elicited ROS production is independent of polyamine oxidation, nitric oxide, and salicylic acid signaling but resembles chemical inhibition of RBOHD (RESPIRATORY BURST OXIDASE HOMOLOG D). Spermine can also suppress ROS elicited by FLS2-independent but RBOHD-dependent pathways, thus pointing to compromised RBOHD activity. Consistent with this, we found that spermine but not putrescine dampens flg22-stimulated cytosolic Ca^2+^ influx. Finally, we found that both polyamines differentially reshape transcriptional responses during PTI and disease resistance to *Pseudomonas syringae*. Overall, we provide evidence for the differential contributions of putrescine and spermine to PTI, with an impact on plant defense.

## Teaser

Putrescine and spermine trigger opposite effects on PAMP-induced ROS production associated with different Ca^2+^signals, ultimately leading to differential regulation of PAMP-induced gene expression and disease resistance to avirulent bacteria.

## Introduction

The most abundant polyamines in plants are the diamine putrescine (Put), the triamine spermidine (Spd), and the tetramine spermine (Spm). The plant contents of polyamines are increased in response to stress. Polyamine levels are regulated through tight control of their biosynthesis, oxidation by polyamine oxidases (PAOs) or copper-containing amine oxidases (CuAOs), conjugation to hydroxycinnamic acids, acylation, and transport (Cona *et al.*, 2006; Alcázar *et al.*, 2010; Tiburcio *et al.*, 2014; Zeiss *et al.*, 2021). Increasing evidence supports the contribution of polyamines to biotic stress resistance, although their effects on defense signaling have not been completely established (Walters, 2003; Tiburcio *et al.*, 2014; Seifi and Shelp, 2019). We recently reported that Put is synthesized in response to systemic acquired resistance (SAR)-inducing bacteria and this polyamine triggers local salicylic acid (SA) accumulation and systemic responses contributing to SAR establishment and defense against *Pseudomonas syringae* (Liu *et al.*, 2020). Studies in Arabidopsis and tobacco indicate that Spm enhances resistance against cauliflower mosaic virus, *Pseudomonas viridiflaba*, *P. syringae*, *Hyaloperonospora arabidopsidis*, *Verticillium dahliae*, and *Botrytis cinerea*. In most cases, Spm responses were found to be dependent on polyamine oxidation (Marina *et al.*, 2008; Moschou *et al.*, 2009; Mitsuya *et al.*, 2009; Sagor *et al.*, 2012; Marco *et al.*, 2014; Mo *et al.*, 2015). Spm also activates the protein kinases SIPK (SA-induced protein kinase) and WIPK (wound-induced protein kinase) in tobacco (Takahashi *et al.*, 2003), as well as mitogen-activated protein kinases (Zhang and Klessig, 1997; Seo *et al.*, 2007), leading to the expression of a number of hypersensitive response marker genes in a reactive oxygen species (ROS)- and Ca^2+^-dependent but SA-independent manner (Takahashi *et al.*, 2004). Overall, the data suggest that Spm contributes to defense through potentiation of the hypersensitive response. However, the potential contribution of Spm to other layers of defense, and pathogen-associated molecular pattern (PAMP)-triggered immunity (PTI) in particular, has not been fully established. *Pseudomonas syringae* produces the small molecule phevamine A, a modified form of Spd that suppresses the potentiating effect of this polyamine on the flagellin-stimulated ROS burst (O’Neill *et al.*, 2018). Therefore, polyamine analogs can be used by pathogens to subvert PTI responses, suggesting the participation of polyamines in the modulation of PTI.

Plants have two layers of pathogen recognition (Dodds and Rathjen, 2010). The first layer is initiated upon the perception of PAMPs by pattern recognition receptors, which leads to PTI. A second intracellular layer relies on nucleotide-binding domain and leucine-rich repeat-containing receptor (NLR) proteins, which directly or indirectly recognize virulence effectors and induce effector-triggered immunity. The Arabidopsis leucine-rich repeat receptor kinase FLS2 (FLAGELLIN SENSITIVE 2) recognizes bacterial flagellin (Gómez-Gómez *et al.*, 2000; Zipfel, 2014). Binding of the immunogenic flagellin peptide (flg22) initiates several downstream responses. One of the earliest signaling events after PAMP recognition is a rapid increase in cytosolic Ca^2+^ concentration ([Ca^2+^]_cyt_), ROS generation, and the activation of MAPKs and Ca^2+^-dependent protein kinases (CPKs), ultimately leading to transcriptional and metabolic reprogramming (Boller and Felix, 2009; Segonzac and Zipfel, 2011). Ca^2+^ is a ubiquitous second messenger whose signal specificity is explained by the duration, amplitude, frequency, and spatial distribution of the Ca^2+^ burst. Specific Ca^2+^ signatures are decoded by Ca^2+^-binding proteins that translate this information into changes in the phosphorylation status of proteins and transcriptional responses (Dodd *et al.*, 2010). ROS have been proposed to act as an antimicrobial agent, facilitate cell wall modifications, and act in local and systemic defense signaling (Lamb and Dixon, 1997; Suzuki *et al.*, 2011; Nathan and Cunningham-Bussel, 2013). ROS are generated by different enzymatic complexes, including Class III peroxidases, oxalate oxidases, lipoxygenases, quinone reductases, amine oxidases including CuAO and PAO, and NADPH oxidases (Cona *et al.*, 2006; Miller *et al.*, 2010). Polyamine oxidation is a source of ROS due to the release of hydrogen peroxide (H_2_O_2_) in the reactions catalyzed by CuAO and PAO. Arabidopsis CuAOs, which localize to the apoplast and peroxisomes, show high affinity for oxidizing Put and much lower affinity for Spd and Spm (Moschou *et al.*, 2012; Planas-Portell *et al.*, 2013). Arabidopsis PAOs, which are found in the cytosol and peroxisomes, use Spd and Spm as preferential substrates and catalyze back-conversion reactions that reverse the polyamine biosynthetic pathway (Alcázar *et al.*, 2010; Moschou *et al.*, 2012; Tiburcio *et al.*, 2014). ROS production during PTI is predominantly dependent on the NADPH oxidase RBOHD (RESPIRATORY BURST OXIDASE HOMOLOG D) (Nühse *et al.*, 2007; Zhang *et al.*, 2007). In general, RBOH proteins transfer electrons from cytosolic NADPH or NADH to apoplastic oxygen, producing superoxide anion (O_2_^–^), which can be converted to H_2_O_2_ by superoxide dismutases (Marino *et al.*, 2012; Suzuki *et al.*, 2012). RBOHs have Ca^2+^-binding EF-hand motifs in their N-terminal region that bind Ca^2+^. Indeed, Ca^2+^ binding is important for the regulation of RBOHD, since treatment with Ca^2+^ chelators and point mutations in EF-hand motifs compromise PAMP-triggered ROS production (Kadota *et al*., 2004, 2014; Ogasawara *et al.*, 2008; Ranf *et al.*, 2011; Segonzac and Zipfel, 2011; Kimura *et al.*, 2012). ROS produced by RBOHD activity also induce Ca^2+^ influx, thus suggesting a positive feedback regulation that boosts ROS production (Ranf *et al.*, 2011). Other mechanisms of RBOHD regulation involve phosphorylation at different sites by the protein kinase BIK1 (BOTRYTIS-INDUCED KINASE1) and CPKs upon PAMP perception (Boudsocq *et al.*, 2010; Suzuki *et al.*, 2011; Marino *et al.*, 2012; Dubiella *et al.*, 2013; Li *et al.*, 2014; Kadota *et al.*, 2014). RBOHD phosphorylation by BIK1 is independent of calcium-based regulatory mechanisms, but Ca^2+^ is required for the ultimate PAMP-triggered RBOHD activation (Kadota *et al.*, 2014). In addition, RBOHs are also regulated by binding of small GTPases, 14-3-3 proteins, phosphatidic acid, and *S*-nitrosylation (Morel *et al.*, 2004; Elmayan *et al.*, 2007; Wong *et al.*, 2007; Zhang *et al.*, 2009; Yun *et al.*, 2011). The many regulatory mechanisms, as well as the broad range of functions of RBOH family members in stress and development, suggest their participation as molecular hubs mediating ROS signaling.

In this work, we investigated the effect of polyamines on the PAMP-elicited ROS burst, which is one of the earliest PTI responses. By focusing on flg22 elicitation of PTI, we found that Spm strongly inhibits flg22-mediated ROS production, whereas Put exhibited the opposite effect. Through genetic and pharmacological approaches, we provide evidence that the inhibitory effect of Spm on the flg22-triggered ROS burst is independent of polyamine oxidation, nitric oxide (NO) signaling, and the defense components EDS1 (ENHANCED DISEASE SUSCEPTIBILITY 1), PAD4 (PHYTOALEXIN DEFICIENT 4), SA, and NPR1 (NONEXPRESSER OF PR GENES 1), and cannot be ameliorated by Put treatment. Inhibition of ROS production by Spm is also observed in response to FLS2-independent but RBOHD-dependent ROS-inducing agents such as methyl viologen (MV). Spm mimics the effect of Ca^2+^ chelators and Ca^2+^ channel blockers that compromise RBOHD-dependent ROS production in response to flg22. In agreement with this, we found that Spm, but not Put, dampens the flg22-triggered Ca^2+^ influx required for RBOHD activation. These polyamines also differentially reshape the transcriptional responses to flg22 and PAMP-mediated disease resistance against *P. syringae*. Overall, we provide evidence for the differential contributions of Put and Spm to PTI signaling, with an impact on plant defense.

## Materials and methods

### Plant materials

Seeds of the different genotypes were directly sown on soil (40% peat moss, 50% vermiculite, and 10% perlite). Seeds were stratified in the dark at 4 °C for 2–3 days to stimulate germination. The different plant genotypes were grown at 20–22 °C under 12 h light/12 h dark photoperiod cycles. Genotypes used in this work were obtained from the Eurasian Arabidopsis Stock Center (https://arabidopsis.info/) or were previously described: *adc1-3*, *adc2-4*, and *spms* (Alcázar *et al.*, 2006; Cuevas *et al.*, 2008; Liu *et al.*, 2019), *eds1-2* (Feys *et al.*, 2005), *pad4-1* (Glazebrook *et al.*, 1997), *sid2-1* (Wildermuth *et al.*, 2001), *npr1-1* (Cao *et al.*, 1997), *fls2* (Heese *et al.*, 2007), *rbohd* N663633 (SALK_109396C), *rbohd* N670541 (SALK_035391C), *rbohf* N657584 (SALK_034674C), *rbohd/f* N9558 (CS9558), *atao1* N672056 (SALK_127609C), *cuao1* N608014 (SALK_108014), *cuao2* N677606 (SALK_012167C), *cuaoα1* N661128 (SALK_125537C), *cuaoα2* N677690 (SALK_037584C), *cuaoδ* N686526 (SALK_094630C), *cuaoε1* N670103 (SALK_124509C), *cuaoε2* N730426 (GK-422D03.08), *cuaoγ2* N2054517 (GK-051A08.10), *pao1* N658095 (SALK_013026C), *pao2* N660420 (SALK_049456C), *pao3* N668943 (SALK_121288C), *pao4* N653495 (SALK_133599C), and *pao5* N679676 (SALK_053110C). The *nia1 nia2 noa1-2* triple mutant described by Lozano-Juste and León (2010) was kindly provided by Prof. José León (Instituto de Biología Molecular y Celular de Plantas, Spain).

### Flg22-elicited ROS measurements

The detection and quantitation of flg22-elicited ROS was performed by monitoring luminescence using a 96-well microplate luminometer (Luminoskan, Thermo Fisher Scientific). Leaf discs (0.5 cm diameter) from fully expanded leaves of 5-week-old plants were incubated for 24 h in 200 µl sterile water. The water was then replaced with a solution containing 10 μg ml^–1^ horseradish peroxidase (Merck), 100 µM of the luminol derivative L-012 (Wako Chemicals) and the different treatments. For pre-incubation assays, leaf discs were incubated with Put (100 µM) or Spm (100 µM) for 24 h before flg22 (1 µM) elicitation. A minimum of 12 replicates per genotype and treatment were used in each analysis. Photon counts (expressed as relative light units) were determined every 2 min in each replicate. Total photon counts were obtained by summing all photon counts over the time of analysis.

### Determination of free polyamine concentrations

The concentrations of free Put, Spd, and Spm were determined by high-performance liquid chromatography separation of dansyl chloride-derived polyamines as described by Liu *et al.* (2020). Analyses were performed in three biological replicates per treatment, each including three technical replicates.

### Pathogen infection assays

*Pseudomonas syringae* pv. *tomato* DC3000 (*Pst* DC3000) was inoculated into fully expanded leaves of 5-week-old plants by syringe infiltration using a bacterial suspension (OD_600_=0.005) in 10 mM MgCl_2_. The number of *Pst* DC3000 colony-forming units per cm^2^ leaf area was determined at 72 h post-inoculation as described by Liu *et al.* (2020), using eight biological replicates per treatment and genotype.

### Pharmacological treatments

Leaf discs (0.5 cm diameter) from fully expanded leaves of 5-week-old plants were incubated at room temperature for 24 h in 200 µl sterile water. The water was then replaced with a solution containing the different pharmacological treatments (as described below) and further incubated for 3 h at room temperature. After the incubation, flg22 was added to a final concentration of 1 µM and flg22-elicited ROS production was detected by monitoring luminescence as described above. The pharmacological treatments and concentrations used were as follows: 5 mM dimethylthiourea (DMTU), 5 mM 2-bromoethylamine hydrobromide (BEA), 20 µM diphenyleneiodonium chloride (DPI), 1 mM reduced l-glutathione (GSH), 100 µM 2-4-carboxyphenyl-4,4,5,5-tetramethylimidazoline-1-oxyl-3-oxide (cPTIO), 2 mM EGTA, 1 mM lanthanum chloride (LaCl_3_), 50 µM or 300 µM cycloheximide (CHX), 20 µM latrunculin B (Lat B), and 2.5 µM brassinazole (BRZ).

### DAB and trypan blue staining

3,3ʹ-diaminobenzidine (DAB) staining was performed by incubation of leaves in 3,3ʹ-diaminobenzidine tetrahydrochloride (1 mg ml^–1^, pH 3.8) overnight followed by destaining in 100% ethanol for 3 h (Clarke, 2009). Trypan blue staining for cell death visualization was performed as previously described (Alcázar *et al.*, 2009).

### Ca^2+^ measurements

A transgenic (Col-0) line expressing cytosolic apoaequorin was used for the quantitation of [Ca^2+^]_cyt_ (Knight *et al.*, 1991). Leaf discs from 5-week-old plants expressing apoaequorin were incubated in 10 µM coelenterazine for 24 h in the dark in 96-well plates. Afterwards, the liquid was replaced with 100 µl H_2_O. Luminescence was recorded every 2 min during the different treatments, using a microplate luminometer (Luminoskan, Thermo Fisher Scientific). To calculate absolute cytoplasmic Ca^2+^ concentrations, the remaining aequorin present in each replicate was completely discharged by adding 100 µl CaCl_2_ (2 M) in 20% ethanol (Fricker *et al.*, 1999) and photon counts were recorded for a further 20 min. The final [Ca^2+^]_cyt_ was calculated according to Rentel and Knight (2004).

### RNA-seq gene expression analyses

Polyamines (Spm and Put), flg22, and mock (water) treatments were performed in three biological replicates by leaf infiltration of 5-week-old wild-type (Col-0) plants. Infiltrated leaves were collected at 24 h of treatment for total RNA extraction. Total RNA was extracted using TRIzol (Thermo Fisher Scientific) and further purified using a RNeasy kit (Qiagen) according to the manufacturer’s instructions. Total RNA was quantified in a Qubit fluorometer (Thermo Fisher Scientific) and checked for purity and integrity in a Bioanalyzer-2100 device (Agilent Technologies). RNA samples were further processed by the Beijing Genomics Institute for library preparation and RNA sequencing using DNBSEQ. Libraries were prepared using the MGIEasy RNA Library Prep kit (MGI Tech) according to the manufacturer’s instructions and each library was paired-end sequenced (2 × 100 bp) on DNBSEQ-G400 sequencers. Read mapping and expression analyses were performed using the CLC Genomics Workbench 21 version 21.0.5 (Qiagen). Only significant expression differences (fold change≥2; Bonferroni-corrected *P*-value≤0.05) were considered. Principal component analysis (PCA), hierarchical clustering analysis (HCA) and Gene Ontology (GO) analyses were performed using the CLC Genomics Workbench 21 version 21.0.5 (Qiagen) and the Gene Ontology resource (http://geneontology.org) using annotations from Araport11 (Cheng *et al.*, 2017; Carbon *et al.*, 2019). Pathway enrichment analyses were performed using PLANTCYC 15.0.1 (https://plantcyc.org/) (Hawkins *et al.*, 2021) and KEGG pathway analyses (https://www.genome.jp/kegg/) (Kanehisa and Goto, 2000).

### qRT–PCR gene expression analyses

Total RNA was extracted using TRIzol reagent (Thermo Fisher Scientific). RNA (2 μg) was treated with DNAse I (Thermo Fisher Scientific) and first-strand cDNA was synthesized using Superscript IV reverse transcriptase (Thermo Fisher Scientific) and oligo(dT) according to the manufacturer’s instructions. Quantitative real-time PCR using the SYBR Green I dye method was performed on a Roche LightCycler 480 II detector system with the following PCR conditions: 95 °C for 2 min, followed by 40 cycles of 95 °C for 15 s, 60 °C for 30 s, and 68 °C for 20 s. Standard curves were performed for quantification. Gene expression was normalized using *ACTIN2* (*At3g18780*) and *UBQ10* (*At4g05320*) as housekeeping genes. Primer sequences used for gene expression analyses were previously reported: *WRKY22*, *WRKY29*, *FRK1*, *NHL10*, and *ACTIN2* (Liu *et al.*, 2019), and *UBQ10* (Alcázar *et al.*, 2014). The qRT–PCR analyses were always performed on at least three biological replicates, each with three technical replicates.

## Results

### Effect of Put and Spm on the flg22-triggered ROS burst

**Fig. 1.**
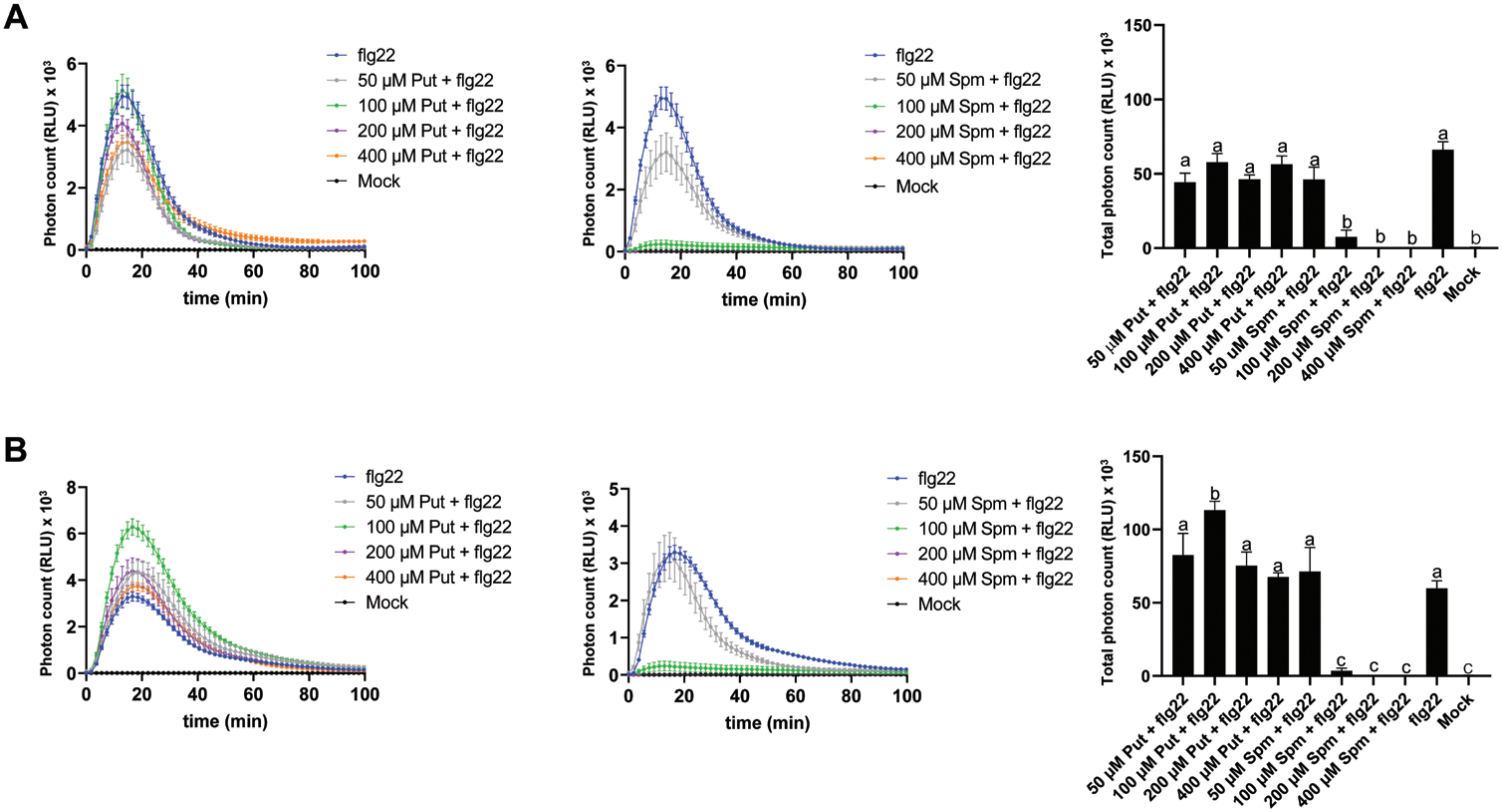
Effect of Put and Spm on the flg22-elicited ROS burst. (A) Leaf discs from fully expanded leaves of 5-week-old wild-type (Col-0) plants were treated with flg22 (1 µM) and Put or Spm at the indicated concentrations (50–400 µM). (B) Leaf discs were pre-incubated with Put or Spm at the indicated concentrations 24 h before treatment with flg22 (1 µM) to elicit ROS. Values represent the mean ±SE from at least 12 replicates per treatment and are expressed in photon counts [relative light units (RLU)]. Different letters indicate values that are significantly different (*P*<0.05) according to Tukey’s HSD test.

To study the effect of different polyamines on PTI, we first analyzed the contribution of Put and Spm to the flg22-triggered ROS burst in Arabidopsis. ROS production was measured in wild-type plants treated with flg22 (1 µM) supplemented with different concentrations of Put and Spm (50, 100, 200, and 400 µM) or mock treated (Fig. 1). Co-treatments consisting of flg22 with Put produced no significant changes in the flg22-triggered ROS burst (Fig. 1A). However, pre-incubation with Put (100 µM) 24 h before flg22 elicitation triggered higher ROS production compared with mock pre-treatment (Fig. 1B). In contrast, concentrations of Spm of 100 µM and higher strongly inhibited flg22-triggered ROS production (Fig. 1A, 1B). Co-treatment consisting of flg22 with Put and Spm also led to inhibition of the ROS burst (Supplementary Fig. S1). The inhibitory effect of Spm on the flg22-triggered ROS burst was also evident in the SA-related defense mutants *eds1-2*, *pad4-1*, *sid2-1*, and *npr1-1* (Supplementary Fig. S2), pointing to an EDS1/PAD4, SA and NPR1-independent response.

Incubation with the individual polyamines (Put or Spm) at different concentrations resulted in much lower but sustained apoplastic ROS production, which showed a plateau between 4 h and 8 h of the 100 µM Put or 100 µM Spm treatments (Supplementary Fig. S3A). On the other hand, flg22 (1 µM) triggered a significant accumulation of Put and dampened Spm content at 24 h of treatment in wild-type plants (Supplementary Fig. S3B). The polyamine-triggered ROS production kinetics did not overlap with the flg22-elicited ROS production response. Interestingly, Spm-triggered ROS production was abrogated in *rbohd* but not *rbohf* mutants (Supplementary Fig. S4). The results indicated that the increase in apoplastic ROS stimulated by Spm is mainly derived from RBOHD activity. Since RBOHD is sensitive to redox perturbations (Torres *et al.*, 2005), the effect of Spm on the stimulation of RBOHD activity might be due to a Spm-triggered intracellular ROS imbalance.

### Flg22-triggered ROS burst in *adc1*, *adc2*, and *spms* mutants

**Fig. 2.**
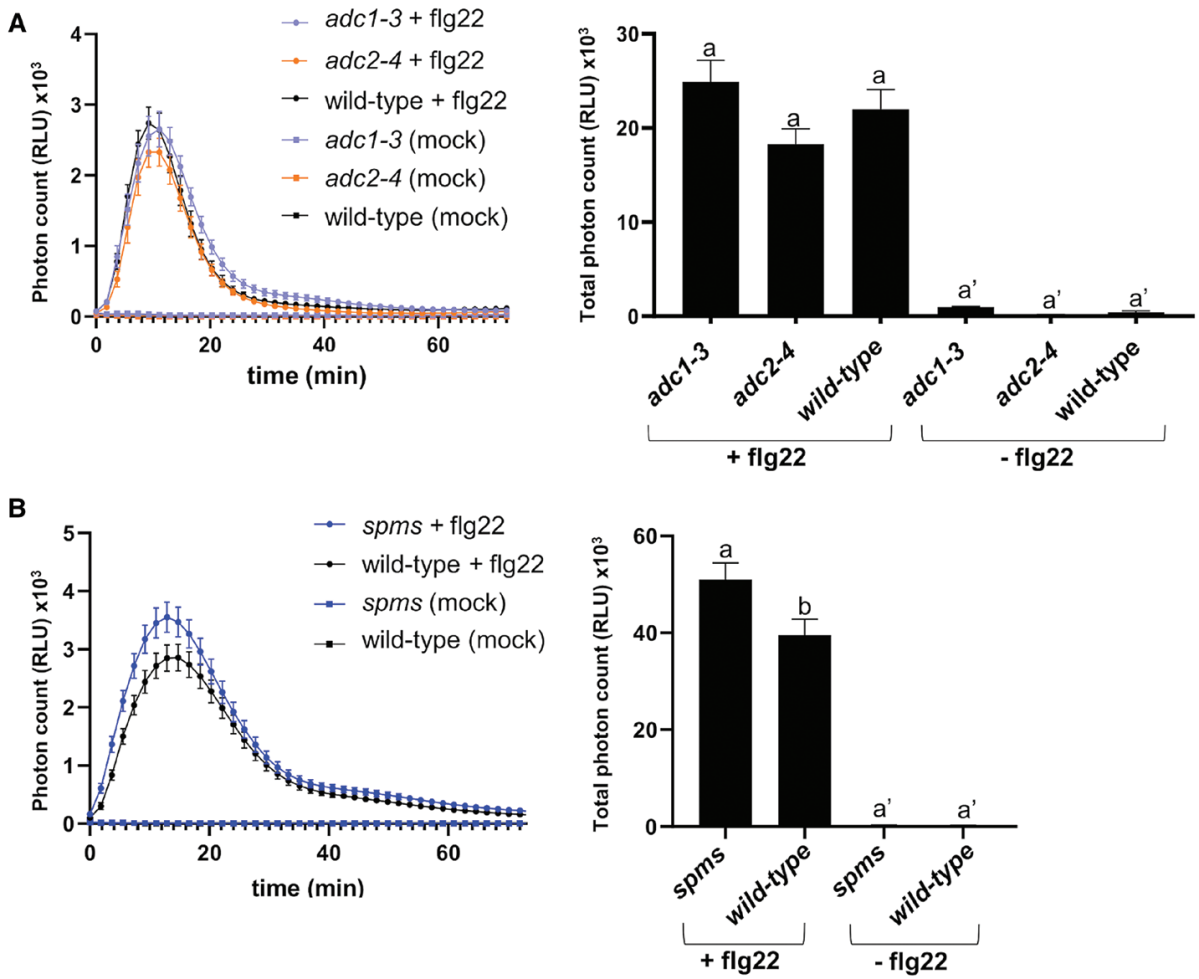
Flg22-stimulated ROS burst in (A) *adc1-3*, *adc2-4*, and (B) *spms* mutants. Leaf discs from fully expanded leaves of 5-week-old wild-type (Col-0) plants and mutants were treated with flg22 (1 µM) or mock (water). Values represent the mean ±SE from at least 12 replicates per treatment and are expressed in photon counts [relative light units (RLU)]. Different letters indicate values that are significantly different (*P*<0.05) according to Tukey’s HSD test.

To further study the effect of Put and Spm on the flg22-triggered ROS burst, we used the *arginine decarboxylase 1* (*adc1-3*) and *arginine decarboxylase 2* (*adc2-4*) mutants compromised in Put biosynthesis, and the *spermine synthase* (*spms*) mutant deficient in Spm biosynthesis (Fig. 2). The *adc1-3* and *adc2-4* mutants showed similar flg22-triggered ROS production to that of the wild type (Fig. 2A). In contrast, flg22-triggered ROS levels were significantly higher in *spms* than in wild-type plants (Fig. 2B). The data were consistent with an inhibitory effect of Spm on the flg22-elicited ROS burst (Fig. 1). We concluded that Put and Spm have opposite effects on flg22-triggered ROS production, with Put increasing and Spm lowering the amplitude of the ROS response.

### Effect of Put and Spm on flg22-triggered *Pst* DC3000 disease resistance

**Fig. 3.**
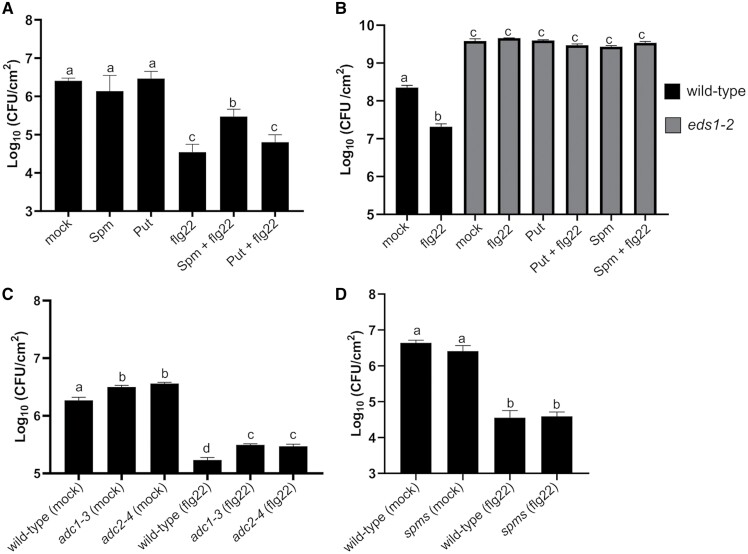
Analysis of *Pst* DC3000 disease resistance in (A) wild-type plants and (B) *eds1-2* mutant plants pre-infiltrated with mock (10 mM MgCl_2_), Put (100 µM), Spm (100 µM), flg22 (1 µM), or combinations (Spm+flg22, Put+flg22). (C, D) Disease resistance phenotypes to *Pst* DC3000 in (C) *adc1-3*, *adc2-4* and (D) *spms* mutants pre-infiltrated with flg22 (1 µM) or mock (10 mM MgCl_2_). Pre-infiltrations were performed 24 h before *Pst* DC3000 inoculation. In all treatments, fully expanded leaves of 5-week-old Arabidopsis plants were infiltrated with *Pst* DC3000 (OD_600 nm_=0.005). Bacterial numbers were assessed at 72 h post-inoculation and expressed as colony-forming units (CFU) per cm^2^ leaf area. Values are the mean ±SD from at least eight biological replicates. Different letters indicate values that are significantly different (*P*<0.05) according to Tukey’s HSD test.

Pre-treatment of wild-type plants with flg22 induces resistance to *Pst* DC3000 (Zipfel *et al.*, 2004). Given the opposite effects of Put and Spm on the flg22-triggered ROS burst, we determined the *Pst* DC3000 disease resistance phenotypes in wild-type plants and *eds1-2* mutant plants treated with flg22 (1 µM), Put (100 µM), Spm (100 µM), combinations (100 µM Put+1 µM flg22; 100 µM Spm+1 µM flg22), and mock treatment (Fig. 3). Wild-type plants pre-infiltrated with (Spm+flg22) supported higher bacterial growth than those pre-treated with flg22 or (Put+flg22) (Fig. 3A). These results were consistent with the observed inhibition of the flg22-triggered ROS burst by Spm, which could partly compromise flg22-elicited defenses (Figs 1, 2). In contrast, pre-treatment of wild-type plants with the individual polyamines (100 µM) did not lead to significant changes in *Pst* DC3000 disease resistance compared with mock treatment (Fig. 3A). The data indicated that Spm is not a non-specific suppressor of defense responses. No differences were observed in bacterial growth between the different treatments in the *eds1-2* mutant (Fig. 3B).

Although we did not detect increased disease resistance to *Pst* DC3000 after treatment with 100 µM Put, higher concentrations of Put (200–500 µM) caused lower bacterial growth in the wild type (Supplementary Fig. S5), which otherwise did not correlate with the amplitude of the flg22-triggered ROS burst (Fig. 1B). The *adc1-3* and *adc2-4* mutants were more susceptible to infection by *Pst* DC3000 than wild-type plants in both flg22-pre-infiltrated and mock-treated conditions (Fig. 3C). The data supported a positive contribution of Put to defense independent of FLS2 signaling. Infiltration of the *spms* mutant with *Pst* DC3000 led to similar bacterial growth to that observed in the wild type. In addition, no significant differences in *Pst* DC3000 growth were observed between wild-type and *spms* plants pre-treated with flg22 (Fig. 3D). This indicated that the enhanced flg22-triggered ROS burst in *spms* (Fig. 2B) did not translate into higher disease resistance. Due to the striking inhibitory effect of Spm on the flg22-triggered ROS burst, we focused on this polyamine in further analyses.

### Contribution of polyamine oxidation to the Spm-mediated inhibition of the flg22-triggered ROS burst

Many defense-related traits attributed to polyamines are associated with ROS production derived from polyamine oxidation. To determine whether polyamine oxidation by amine oxidases (CuAO and PAO) is necessary for the inhibitory effect of Spm on the flg22-triggered ROS burst, we used *cuao* (*atao1*, *cuao1*, *cuao2*, *cuaoα1*, *cuao*α2, *cuao*δ, *cuao*ε1, *cuao*ε2, *cuao*γ2) and *pao* (*pao1–pao5*) loss-of-function mutants to study their ROS production in response to flg22 and (Spm+flg22). The different *cuao* and *pao* mutants did not show significant differences in flg22-triggered ROS levels compared with the wild type (Supplementary Fig. S6A). In addition, the different amine oxidase (CuAO and PAO) mutations or treatment with the amine oxidase inhibitor BEA did not rescue the inhibitory effect of Spm on the flg22-elicited ROS burst (Supplementary Figs S6B, S7). The data suggested that polyamine oxidation is dispensable for the inhibitory effect of Spm on the flg22-elicited ROS burst.

### Spm inhibits RBOHD-dependent ROS production

The inhibition of the flg22-triggered ROS burst by Spm was mimicked by treatments with the NADPH oxidase inhibitor DPI, the ROS scavengers DMTU and GSH, the Ca^2+^ chelator EGTA, and the Ca^2+^ channel blocker LaCl_3_ (Supplementary Fig. S7). To further study the inhibitory effect of Spm on the flg22-triggered ROS burst, we used a pharmacological approach with wild-type plants treated with the NO scavenger cPTIO, the protein synthesis inhibitor CHX, the actin depolymerization inhibitor Lat B, which strongly reduces flg22-induced FLS2 internalization (Robatzek *et al.*, 2006), and the brassinosteroid (BR) biosynthesis inhibitor BRZ (Supplementary Figs S7, S8). Brassinolides have also been shown to inhibit FLS2 signaling, including the flg22-elicited ROS burst in Arabidopsis (Albrecht *et al.*, 2012; Belkhadir *et al.*, 2012).

Treatments with cPTIO, Lat B, or BRZ did not rescue flg22-triggered ROS production in the presence of Spm (Supplementary Figs S7, S8A). In addition, the inhibitory effect of Spm on the flg22-elicited ROS burst was not compromised in the NO-deficient *nia1 nia2 noa1-2* triple mutant (Supplementary Fig. S8B). Therefore, the inhibitory effect of Spm on the flg22-triggered ROS burst is not mediated by NO, and is not due to FLS2 internalization or *de novo* BR biosynthesis (Albrecht *et al.*, 2012; Belkhadir *et al.*, 2012). Treatment with CHX did not compromise the inhibition of the flg22-triggered ROS burst by Spm, which indicated that this effect does not require *de novo* protein biosynthesis. Interestingly, CHX treatment produced very high amounts of ROS, which were absent in (CHX+Spm) treatments (Supplementary Figs S7, S8C). CHX-triggered ROS production was also compromised in *rbohd* but not *fls2*, which supported the dependence of CHX-triggered ROS production on RBOHD independent of FLS2 (Supplementary Fig S8C, D). The data suggested that Spm inhibits flg22-triggered ROS production through inhibition of RBOHD activity and/or ROS scavenging capacity.

### Analysis of the ROS scavenging and cell death trigger capacity of Spm

**Fig. 4.**
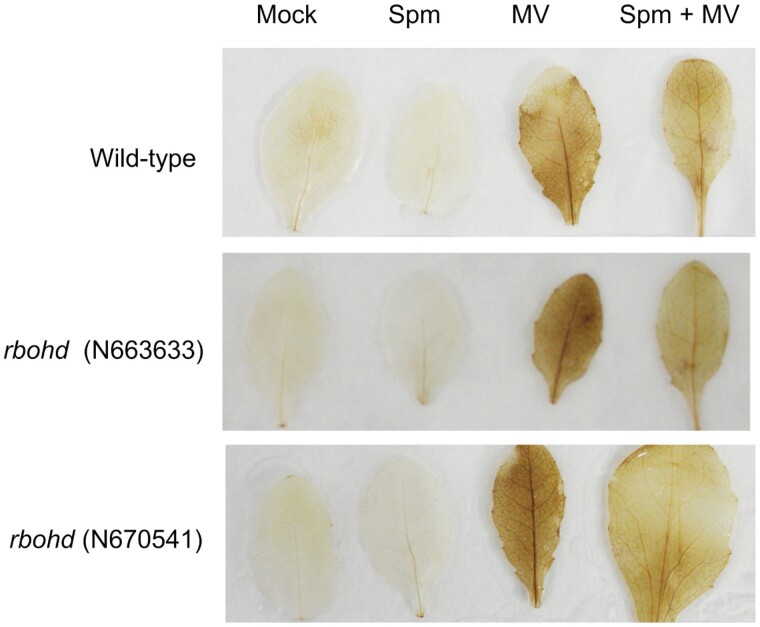
3,3ʹ-diaminobenzidine staining of wild-type and *rbohd* mutants infiltrated with Spm (100 µM), methyl viologen (MV) (100 µM), or both (100 µM Spm+100 µM MV). Staining was performed at 24 h of treatment.

To investigate the potential ROS-scavenging capacity of Spm in plants, we performed DAB staining in wild-type and *rbohd* leaves infiltrated with Spm (100 µM), the ROS producer MV (100 µM), Spm (100 µM)+MV (100 µM), or mock treated (Fig. 4). Infiltration with MV led to uniform DAB precipitates in wild-type and *rbohd* leaves, indicative of high H_2_O_2_ production independent of RBOHD. In contrast, infiltration with Spm (100 µM) did not lead to evident DAB staining and resembled the mock treatment. Co-infiltration of MV+Spm only partly alleviated the presence of DAB precipitates, which otherwise were still evident in wild-type and *rbohd* leaves (Fig. 4). MV, Spm, or MV+Spm did not induce cell death in any of the genotypes tested, as revealed by trypan blue staining (Supplementary Fig. S9). The data indicated that Spm (100 µM) only partly scavenges ROS production in Arabidopsis leaves and, at this concentration, is not a cell death trigger.

### Effect of Spm on flg22-induced Ca^2+^ influx

**Fig. 5.**
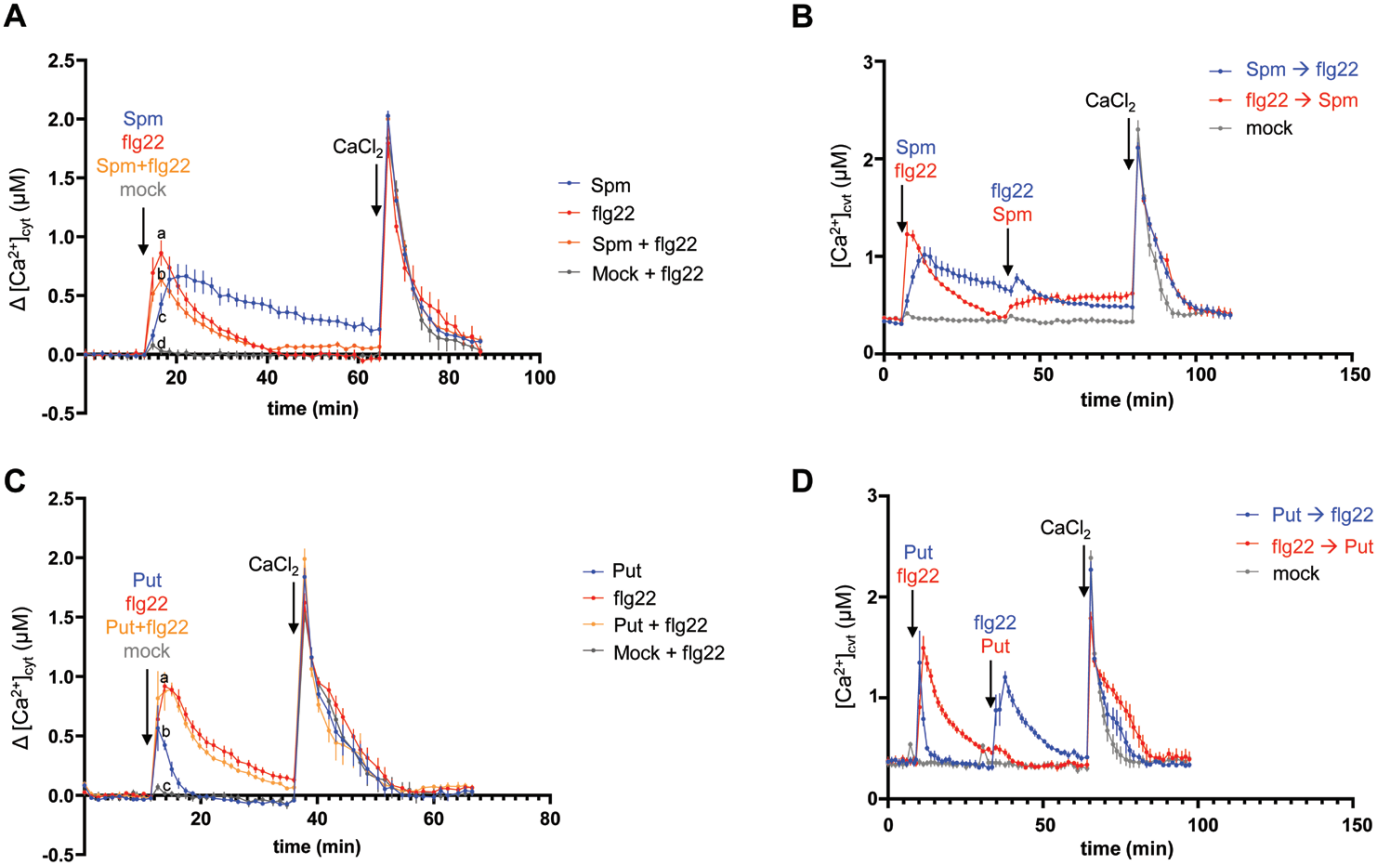
Flg22, Put, and Spm-induced Ca^2+^ signatures. Leaf discs of an apoaequorin-expressing line (Knight *et al.*, 1991) were (A) treated with 1 µM flg22, 100 µM Spm, 100 µM Spm+1 µM flg22, or mock treated (water); (B) pre-incubated with 100 µM Spm or 1 µM flg22 at the indicated time before flg22 (1 µM) or Spm (100 µM) elicitation, respectively; (C) treated with 1 µM flg22, 100 µM Put, 100 µM Put+1 µM flg22, or mock treated (water); or (D) pre-incubated with 100 µM Put or 1 µM flg22 at the indicated time before flg22 (1 µM) or Put (100 µM) elicitation, respectively. Data represent Δ[Ca^2+^]_cyt_ after normalization to steady-state [Ca^2+^]_cyt_ prior to polyamine or flg22 elicitation, or absolute [Ca^2+^]_cyt_ (µM). Values were obtained from at least six replicates per treatment. Each point represents the mean ±SE. Different letters indicate values that are significantly different (*P*<0.05) according to Tukey’s HSD test. This experiment was repeated twice with similar results.

PAMPs induce a rapid and transient increase of [Ca^2+^]_cyt_ by the influx of Ca^2+^ from the extracellular environment or internal stores (McAinsh and Pittman, 2009). This Ca^2+^ burst operates upstream of many PAMP-elicited responses and is necessary for RBOHD activity (Boller and Felix, 2009; Segonzac and Zipfel, 2011; Ranf *et al.*, 2011). We used the apoaquorein bioluminescent Ca^2+^ sensor to measure the steady-state levels and dynamics of [Ca^2+^]_cyt_ in response to flg22, Spm, Put, and combinations thereof in the wild-type background (Fig. 5). Flg22 treatment triggered a rapid Ca^2+^ influx, which was significantly inhibited by co-treatment with Spm (Fig. 5A). The inhibitory effect of Spm on the flg22-triggered [Ca^2+^]_cyt_ influx was more evident in plants pre-incubated with Spm before the flg22 challenge (Fig. 5B). Spm also triggered significant increases in [Ca^2+^]_cyt_ (Fig. 5A) that were attenuated by flg22 pre-treatment (Fig. 5B).

Put also triggered [Ca^2+^]_cyt_ elevation, although the amplitude of the Ca^2+^ signal was much lower than with Spm (Fig. 5C). Flg22-triggered increases in [Ca^2+^]_cyt_ were not significantly affected by co-treatments or pre-treatments with Put (Fig 5C, D). We concluded that Put and Spm trigger different Ca^2+^ signals that may contribute to polyamine specificity. Furthermore, we showed that Spm compromises the Ca^2+^ influx elicited by flg22 that is necessary for RBOHD activation, thus providing a plausible explanation for the inhibitory effect of Spm on flg22-triggered ROS production beyond its ROS-scavenging capacity.

### Effect of Spm and Put on flg22-elicited transcriptional responses

To further investigate the differential effect of Put and Spm on the modulation of flg22-triggered responses, we determined global changes in expression at 24 h of Put (100 µM), Spm (100 µM), flg22 (1 µM), Spm (100 µM)+flg22 (1 µM), Put (100 µM)+flg22 (1 µM), and mock treatments in wild-type plants. The RNA-seq data were used for PCA and HCA (Supplementary Fig. S10; Supplementary Tables S1.1–S1.5, S2.1–S2.19, S3.1–S3.19). The principal component 1 (PC1) of the PCA explained 32% of total variance and mainly differentiated between flg22-treated and flg22-untreated samples (Supplementary Fig. S10A). Flg22 treatment also discriminated between the two major clades of the HCA analysis (Supplementary Fig. S10B). The principal component 2 (PC2) (16.1% of total variance) revealed the differences in expression due to the polyamine treatments (Supplementary Fig. S10A), which also grouped into separate subclades of the HCA analysis (Supplementary Fig. S10B).

A total of 554 and 368 genes were significantly deregulated (fold-change≥2.0 and Bonferroni-corrected *P*-value≤0.05) after 100 µM Put or 100 µM Spm treatments, respectively (Supplementary Fig. S11; Supplementary Tables S1.1, S1.2). Among the 396 genes deregulated only by Put (Supplementary Table S1.3), we found a significant enrichment in up-regulated genes related to translation, and down-regulated genes related to stress (Supplementary Fig. S11). A low correlation was found between Put and Spm treatments in the set of Put-only deregulated genes (*r*^2^=0.4149) (Supplementary Fig. S11). On the other hand, in the set of 210 genes deregulated only by Spm (Supplementary Table S1.4), up-regulated genes were enriched in hormone, lipid and cytokinin responses, while those down-regulated included GO terms related to the regulation of transcription factor activity, senescence, and biotic responses (Supplementary Fig. S11). Spm-only-deregulated genes also showed a low correlation with Put treatment (*r*^2^=0.6305) (Supplementary Fig. S11). The data were consistent with a differential regulation of gene expression by these two polyamines at 24 h of treatment. Indeed, only 158 genes were commonly deregulated by Put and Spm (Supplementary Fig. S11; Supplementary Table S1.5). Overexpressed genes within this gene expression sector were mainly related to cell wall biogenesis and cell wall organization (Supplementary Fig. S11). Overall, Put showed a transcriptional effect on ribosome biogenesis that was not evident in the Spm treatment at 24 h of treatment. The polyamines also showed contrasting effects on the expression of genes involved in primary metabolism and transcription factor families. However, both polyamines enhanced the expression of genes encoding enzymes that modify the composition and assembly of the cell wall (Supplementary Fig. S11).

### Comparative gene expression analysis of wild-type plants treated with (Spm+flg22) and flg22

**Fig. 6.**
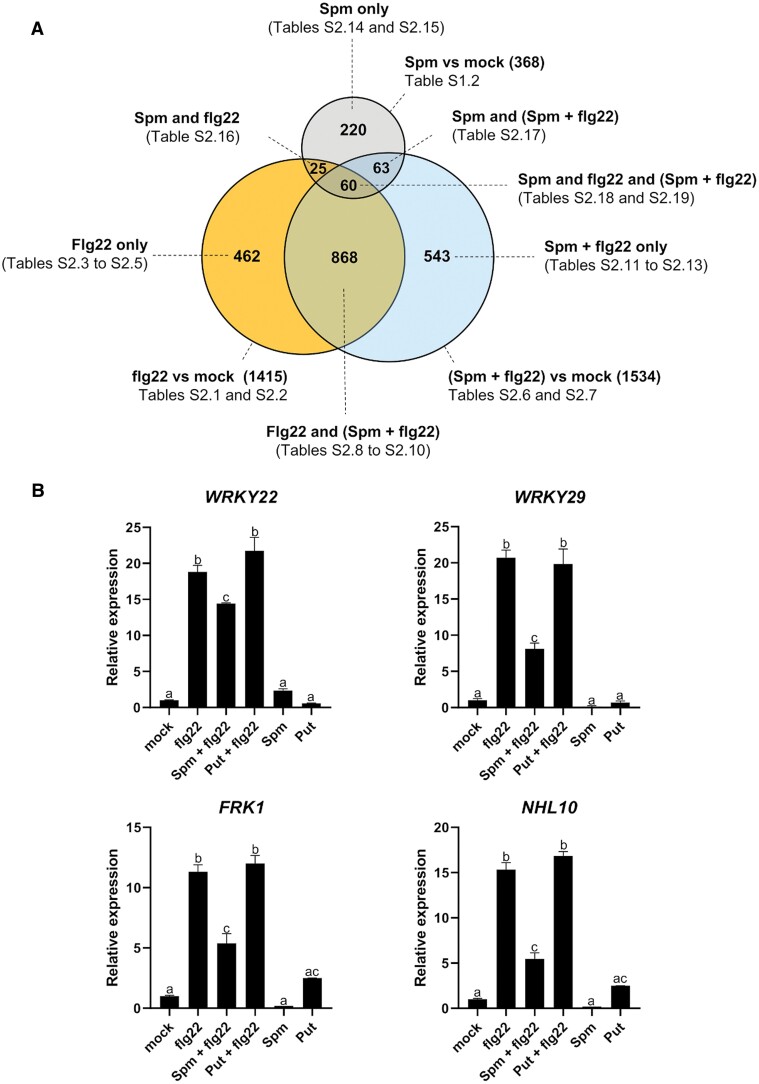
Comparative gene expression analysis of wild-type plants treated with flg22 and Spm+flg22. (A) Venn diagram of genes differentially expressed (fold change≥2; Bonferroni-corrected *P*-value≤0.05) in response to flg22 (1 µM), Spm (100 µM), and Spm (100 µM)+flg22 (1 µM). Genes and GO enrichment analyses within each category are listed in Supplementary Tables S2.1–S2.19. (B) qRT–PCR gene expression analyses of the flg22-inducible genes *WRKY22*, *WRKY29*, *FRK1*, and *NHL10* in wild-type plants at 24 h of treatment with flg22 (1 µM), Spm (100 µM), Put (100 µM), Spm (100 µM)+flg22 (1 µM) or Put (100 µM)+flg22 (1 µM). Values represent the mean ±SD. Different letters indicate values that are significantly different (*P*<0.05) according to Tukey’s HSD test.

Compared with the mock treatment, flg22 triggered the deregulation of 1415 genes (Fig. 6A; Supplementary Table S2.1). Most flg22-responsive genes were related to defense but also included genes involved in ribosome biogenesis (Supplementary Tables S2.1, S2.2). Out of the 1415 flg22-responsive genes, 462 (33%) were not significantly deregulated by flg22 in the presence of Spm (Fig. 6A; Supplementary Fig. S12A; Table S2.3). This set of flg22-only genes was enriched in ribosomal proteins (55 genes) and GO terms related to translation (Supplementary Fig. S13; Supplementary Tables S2.3, S2.4). The flg22-only sector also included genes involved in phenylpropanoid biosynthesis (up-regulated), and down-regulated genes related to carbon starvation, biosynthesis of amino acids, glucosinolates, trehalose and fatty acid metabolism, among others (Supplementary Fig. S13; Supplementary Table S2.5). The data suggested that Spm dampens the expression of a subsector of flg22-responsive genes involved in ribosomal biogenesis and stress metabolism adaptation.

Treatment of wild-type plants with (Spm+flg22) triggered the deregulation of 1534 genes (Fig. 6A; Supplementary Table S2.6), of which only 868 (Supplementary Fig. S12B; Supplementary Table S2.8) and 63 (Supplementary Table S2.17) were common with the flg22 and Spm treatments, respectively. The flg22 and (Spm+flg22) common genes were enriched in GO categories related to SAR, defense, SA and jasmonic acid responses, phenylpropanoid metabolism (all up-regulated), plant hormone biosynthesis and starch degradation (down-regulated), among other biological functions (Fig. 6A; Supplementary Fig. S14; Supplementary Tables S2.9, S2.10). The most abundant molecular functions included enzymes (246 genes), transporters (60 genes), transcription factors (55 genes), and ribosomal proteins and rRNA processing enzymes (47 genes) (Supplementary Fig. S14; Supplementary Table S2.8). The suppressive effect of Spm on the flg22-elicited ROS burst was consistent with the lower up-regulation of the flg22-inducible genes *WRKY22*, *WRKY29*, *FRK1*, and *NHL10* in wild-type plants treated with (Spm+flg22) compared with flg22, determined by qRT–PCR (Fig. 6B).

The (Spm+flg22)-only sector included 543 genes whose biological functions were related to defense responses to bacteria, ROS responses, protein phosphorylation, flavonoid biosynthesis (all up-regulated), and transcription regulation (down-regulated), among others (Fig. 6A, Supplementary Figs S12C, S15; Supplementary Tables S2.11–S2.13). This set of (Spm+flg22)-only genes was used to determine correlation coefficients between flg22 and (Spm+flg22) treatments. We found that both treatments were correlated (*r*^2^=0.9036), despite the difference in the number of genes assigned to each sector (Supplementary Fig. S12E). This is probably due to the threshold criteria used to identify differentially expressed genes (fold change≥2 and Bonferroni-corrected *P*-value≤0.05), which overestimated the differences between treatments (Supplementary Fig. S12B; Supplementary Table S2.8). The correlation between the flg22 and (Spm+flg22) treatments in genes within the flg22-only sector was lower (*r*^2^=0.8056) (Supplementary Fig. S12E).

Genes exclusively deregulated by Spm were significantly enriched in GO terms related to cell wall biogenesis (up-regulated) (Fig. 6A; Supplementary Figs S12D, S16; Supplementary Tables S2.14, S2.15), which suggested that polyamines could contribute to cell wall reinforcement and modifications during plant defense. In this case, no strong correlations were detected between the Spm and (Spm+flg22) treatments (*r*^2^=0.5353) (Supplementary Fig. S12E). We concluded that Spm dampens the up-regulation of flg22-responsive genes, genes encoding ribosomal proteins, and genes related to metabolism adaptation during flg22-elicited defenses.

### Comparative gene expression analysis of wild-type plants treated with (Put+flg22) and flg22

**Fig. 7.**
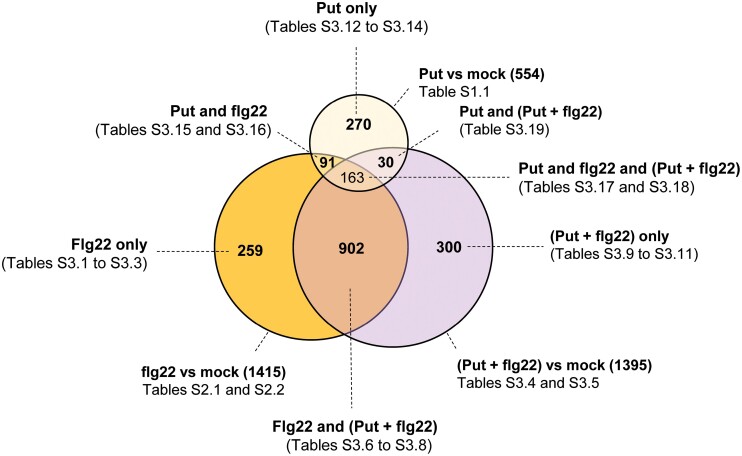
Comparative gene expression analysis of wild-type plants treated with flg22 and Put+flg22. Venn diagram of genes differentially expressed (fold change≥2; Bonferroni corrected *P*-value≤0.05) in response to flg22 (1 µM), Put (100 µM), and Put (100 µM)+flg22 (1 µM). Genes and GO enrichment analyses within each category are listed in Supplementary Tables S3.1–S3.19.

In the Put treatment, out of the 1415 flg22-responsive genes, 259 (18.3%) were not significantly deregulated by flg22 in the presence of Put (flg22-only in Fig. 7 and Supplementary Fig. S17A; Supplementary Table S3.1). However, the flg22 and (Put+flg22) treatments were highly correlated (*r*^2^=0.9439) (Supplementary Fig. S17E), which suggested that the significance threshold also overestimated the differences between the treatments. Flg22-only genes were also enriched in ribosomal proteins (30 genes) (Fig. 7; Supplementary Fig. S18; Supplementary Tables S3.1, S3.2). However, these genes represented almost half of the ribosomal genes detected within the same sector in the comparison with (Spm+flg22) treatment (55 genes) (Fig. 6A; Supplementary Table S2.3). The ‘Put and flg22’ (91 genes) and ‘Put, flg22, and (Put+flg22)’ (163 genes) gene expression sectors were also enriched in GO terms related to translation (Supplementary Tables S3.15–S3.18). This was consistent with the effect of Put on the up-regulation of genes encoding ribosomal proteins and rRNA processing enzymes (Supplementary Fig. S11; Supplementary Table S1.1). Flg22-only up-regulated genes also included enzymes (59) enriched in the phenylpropanoid pathway that were also up-regulated in the common flg22 and (Put+flg22) gene expression sectors (Supplementary Figs S18, S19; Supplementary Tables S3.1–S3.3, S3.6–S3.8).

Treatment of wild-type plants with (Put+flg22) triggered the deregulation of 1395 genes (Fig. 7; Supplementary Table S3.4), of which 902 (Supplementary Fig. S17B; Supplementary Table S3.6) and 30 (Supplementary Table S3.19) were common with the flg22 and Put treatments, respectively. The flg22 and (Put+flg22) common gene expression sector was enriched in GO terms related to SAR, SA signaling, defense, lignin metabolism, ribosome biogenesis, phenylpropanoid metabolism (all up-regulated) and starch catabolism (down-regulated), among other biological functions (Supplementary Fig. S19; Supplementary Tables S3.6–S3.8). The most abundant molecular functions included enzymes (252 genes), transporters (67 genes), transcription factors (53 genes), and others, including 26 ribosomal proteins (Supplementary Fig. S19; Supplementary Table S3.6). In contrast to Spm, the treatment of wild-type plants with (Put+flg22) did not trigger significant changes in the expression of the flg22-inducible genes *WRKY22*, *WRKY29*, *FRK1*, and *NHL10* compared with flg22 treatment alone, as determined by qRT–PCR (Fig. 6B). The data were consistent with the specific effect of Spm on the inhibition of flg22-elicited responses.

The (Put+flg22)-only sector included 300 genes mainly related to defense responses to bacteria (up-regulated), response to oxygen-containing compounds, and response to lipids (down-regulated) (Supplementary Figs S17C, S20; Supplementary Tables S3.9–S3.11). The expression of genes within this sector was also correlated with flg22 treatment (*r*^2^=0.8757), which accounted for quantitative rather than qualitative differences between the sectors (Supplementary Fig. S17E). As in the case of Spm, genes up-regulated only by Put were mostly enriched in cell wall biogenesis functions and showed a low correlation with the other treatments (Fig. 7; Supplementary Figs S17D, S17E, S21; Supplementary Tables S3.12–S3.14).

Collectively, the data indicated that Spm, but not Put, dampens the up-regulation of flg22-inducible genes. In addition, Spm and, less markedly, Put compromise flg22-induced transcriptional up-regulation of genes encoding ribosomal proteins, and both trigger the up-regulation of cell wall biogenesis and modification enzymes that are not deregulated by flg22, thus reshaping the PAMP-induced transcriptional responses.

## Discussion

Spm and its precursors Put and Spd show opposite effects on the stimulation of flg22-elicited ROS production and defense elicitation. While Spm inhibits the flg22-triggered ROS burst (Fig 1A, B), Put (Fig. 1B) and Spd (Supplementary Fig. S22) (O’Neill *et al.*, 2018) potentiate ROS production. Importantly, the inhibitory effect of Spm does not require its oxidation (Supplementary Fig. S6B), is independent of NO signaling (Supplementary Figs S7, S8B), and cannot be compensated for by Put (Supplementary Fig. S1). Put and Spm also show opposite accumulation patterns during PTI. Whereas the Put concentration is increased at 24 h of flg22 elicitation in the wild type, the Spm concentration is decreased (Supplementary Fig. S3B) (Liu *et al.*, 2019). Upon flg22 binding, FLS2 forms a complex with BAK1 (BRI1-ASSOCIATED KINASE1), an LRR-receptor kinase that also serves as co-receptor of BRI1 (BRASSINOSTEROID INSENSITIVE1), which is involved in BR signaling (Zipfel, 2014). BR has also been shown to inhibit FLS2 signaling, including the flg22-elicited ROS burst in Arabidopsis, downstream or independently of the FLS2–BAK1 complex or through competition for BAK1 recruitment by FLS2 and the BR receptor BRI1 (Albrecht *et al.*, 2012; Belkhadir *et al.*, 2012). However, treatment with the BR biosynthesis inhibitor BRZ did not rescue the inhibitory effect of Spm on flg22-triggered ROS production (Supplementary Fig. S8A). In addition, compromised internalization of FLS2 caused by treatment with Lat B did not rescue Spm-triggered ROS inhibition (Supplementary Fig. S7). In addition to the flg22-triggered ROS burst, Spm also inhibited CHX-induced ROS production, which was RBOHD-dependent but FLS2-independent (Supplementary Fig S8C, D). The data suggested that Spm inhibition of flg22-elicited ROS was not due to impaired FLS2 signaling. Rather, Spm mimicked the inhibitory effect of the NADPH oxidase inhibitor DPI, the Ca^2+^ chelator EGTA, and the Ca^2+^ channel blocker LaCl_3_ on flg22-triggered ROS production (Supplementary Fig. S7). Flg22-elicited ROS is mainly produced by RBOHD, whose activation requires Ca^2+^ binding to its N-terminal EF-hand motifs (Kadota *et al*., 2004, 2014; Ogasawara *et al.*, 2008; Segonzac and Zipfel, 2011; Ranf *et al.*, 2011; Kimura *et al.*, 2012). Flg22 treatment triggered a rapid [Ca^2+^]_cyt_ increase that was significantly dampened by Spm but not by Put (Fig. 5). The suppressive effect of Spm on the flg22-triggered Ca^2+^ influx might compromise RBOHD-triggered ROS production, thus being a plausible explanation for the inhibitory effect of Spm on flg22-elicited ROS production.

On the other hand, Spm and Put also triggered the elevation of [Ca^2+^]_cyt_, but the Ca^2+^ signature lasted longer in the treatments with Spm (Fig. 5). The different Ca^2+^ signatures triggered by Put and Spm might contribute to signal specificity, as noted in our RNA-seq analyses (Supplementary Fig. S11).

Rocha *et al.* (2020) reported that Spm was necessary for mucilage production during appressoria morphogenesis in the blast fungus *Magnaporthe oryzae*, by buffering oxidative stress in the endoplasmic reticulum lumen and preventing the unfolded protein response. The antioxidant properties of Spm in plants have been documented (Drolet *et al.*, 1986) and involve the conversion of the polyamine into different adducts, including Spm dialdehyde, in the presence of hydroxyl radicals (Ha *et al.*, 1998). However, the potential ROS scavenging capacity of Spm does not fully explain the inhibitory effect of Spm on flg22-elicited ROS production. We found that Spm (100 µM) shows only partial antioxidant capacity in Arabidopsis leaves infiltrated with MV, a ROS-inducing agent that transfers electrons from photosystem I to molecular oxygen (Fig. 4). In addition, Spm and also its precursor Spd act as ROS scavengers through seemingly similar mechanisms (Khan *et al.*, 1992). However, these two polyamines show opposite effects on flg22-triggered ROS stimulation (Fig. 1; Supplementary Fig. S22) (O’Neill *et al.*, 2018). Furthermore, we found that thermospermine (100 µM), an isomer of Spm, does not inhibit flg22-triggered ROS production (Supplementary Fig. S22). These data indicate that the effect of Spm on flg22-elicited ROS burst inhibition is highly specific.

Polyamines are known to affect ion transport across membranes through intricate mechanisms that are also dependent on polyamine charge (Pottosin and Shabala, 2014). Spm can induce membrane depolarization or hyperpolarization, depending on the concentration at which it is supplied. At low concentration (100 µM), Spm causes weak membrane hyperpolarization and transient efflux of H^+^ but not Ca^2+^. In contrast, Spm at higher concentration (1 mM) causes membrane depolarization in a ROS-independent manner (Pottosin *et al.*, 2014). In contrast to Spm, other polyamines trigger membrane depolarization at any given concentration (Pottosin *et al.*, 2014). ROS derived from polyamine oxidation can also activate a variety of non-selective Ca^2+^-permeable channels leading to increases in [Ca^2+^]_cyt_. Externally supplied polyamines can also trigger NO generation and intracellular Ca^2+^ release through a pathway involving cGMP and cADPR (Pottosin and Shabala, 2014). In addition, polyamines can stimulate Ca^2+^ efflux by the activation of plasma membrane Ca^2+^-ATPase activity (Pottosin *et al.*, 2014). The plasma membrane Ca^2+^-ATPases ACA8 (ARABIDOPSIS-AUTOINHIBITED Ca^2+^-ATPase 8) and ACA10 have been found in complex with FLS2, and the double *aca8 aca10* mutant has been reported to show a decreased flg22-induced Ca^2+^ and ROS burst (Frei dit Frey *et al.*, 2012). However, the identity of the Ca^2+^ channels and detailed mechanisms that mediate the differential Ca^2+^ signature triggered by Put and Spm remain largely unknown.

To further investigate the effect of Spm and Put on PTI, we performed global gene expression analyses in the wild type at 24 h of treatment with flg22, Spm, Put, and combinations (Spm+flg22 and Put+flg22). Despite the inhibitory effect of Spm on the flg22-triggered ROS burst, transcriptional responses to flg22 and *Pst* DC3000 disease resistance were only partly compromised by the polyamine (Figs 3A, 6). Both Put and Spm triggered the up-regulation of genes related to cell wall biogenesis. However, the polyamines exhibited differences in the transcriptional responses of genes related to primary metabolism, transcription factors, and protein synthesis and degradation at 24 h (Supplementary Fig. S11). In a previous study (Liu *et al.*, 2020), we found that polyamines exhibited similar early transcriptional responses at 1 h of treatment. Therefore, there are differences between the early and late transcriptional responses to polyamines. Such differences have also been documented in response to different PAMPs, such as flg22 or oligogalacturonides (Denoux *et al.*, 2008). The flg22, (Spm+flg22), and (Put+flg22) treatments also revealed that polyamines differentially reshape the transcriptional responses to PAMPs. In particular, Spm but not Put compromised the up-regulation of flg22-responsive genes (Fig. 6B). On the other hand, Spm and less markedly Put dampened the flg22-elicited expression of ribosome biogenesis genes (Supplementary Figs S13, S18). Bach-Pages *et al.* (2020, Preprint) found that the RNA-binding activity of eukaryotic initiation factors, elongation factors, and ribosomal proteins was inhibited in response to flg22. The overexpression of genes encoding ribosomal proteins caused by flg22 might be a compensatory mechanism to the PAMP-triggered inhibition of translation. In contrast, the polyamines Spd and Spm, and less markedly Put, are known to stimulate translation elongation and thus protein biosynthesis, which might compensate for the inhibitory effect of flg22 on translation (Igarashi and Kashiwagi, 2015; Dever and Ivanov, 2018).

Collectively, we conclude that polyamines differentially modulate PTI responses including Ca^2+^ signals and ROS production, ultimately leading to changes in global transcriptional responses with an impact on plant defense against *P. syringae*.

## Supplementary data

The following supplementary data are available at *JXB* online.

Fig. S1. Effect of the Put and Spm co-treatment on flg22-elicited ROS burst.

Fig. S2. Effect of Spm on flg22-elicited ROS burst in *eds1-2*, *pad4-1*, *sid2-1*, *npr1-1*, and *fls2* mutants.

Fig. S3. ROS produced by Put and Spm treatments, and free polyamine concentrations in response to flg22.

Fig. S4. Effect of Spm on flg22-elicited ROS burst in *rbohd*, *rbohf*, and double *rbohd/f* mutants.

Fig. S5. Analysis of *Pst* DC3000 disease resistance phenotypes in wild-type plants locally pre-treated with different concentrations of Put.

Fig. S6. Flg22-elicited ROS and effect of Spm on flg22-elicited ROS production in *cuao* and *pao* mutants.

Fig. S7. Pharmacological studies on Spm inhibition of flg22-triggered ROS burst.

Fig. S8. Effect of BRZ, NO, and CHX treatments on Spm inhibition of flg22-triggered ROS burst.

Fig. S9. Trypan blue staining of wild-type and *rbohd* leaves infiltrated with Spm, MV, or both.

Fig. S10. PCA and HCA of RNA-seq gene expression data obtained from wild-type plants treated with Put, Spm, flg22, Put+flg22, Spm+flg22, or mock for 24 h.

Fig. S11. Venn diagram, GO, and expression correlation analyses of genes significantly deregulated in response to Put and Spm at 24 h of treatment in the wild type.

Fig. S12. Mean expression values and correlation analyses of wild-type plants treated with flg22, Spm, and (Spm+flg22).

Fig. S13. Molecular function categorization and metabolic pathway enrichment analysis of genes deregulated by only flg22 compared with Spm and (Spm+flg22) treatments in the wild type.

Fig. S14. Molecular function categorization and metabolic pathway enrichment analysis of genes commonly deregulated in flg22 and (Spm+flg22) treatments in the wild type.

Fig. S15. Molecular function categorization and metabolic pathway enrichment analysis of genes differentially expressed in only (Spm+flg22) compared with flg22 and Spm treatments.

Fig. S16. Molecular function categorization and metabolic pathway enrichment analysis of genes differentially expressed in only Spm compared with flg22 and (Spm+flg22) treatments.

Fig. S17. Mean expression values and correlation analyses of wild-type plants treated with flg22, Put, and (Put+flg22).

Fig. S18. Molecular function categorization and metabolic pathway enrichment analysis of genes deregulated by only flg22 compared with Put and (Put+flg22) treatments.

Fig. S19. Molecular function categorization and metabolic pathway enrichment analysis of genes commonly deregulated in flg22 and (Put+flg22) treatments in the wild type.

Fig. S20. Molecular function categorization and metabolic pathway enrichment analysis of genes deregulated in only (Put+flg22) compared with flg22 and Put treatments.

Fig. S21. Molecular function categorization and metabolic pathway enrichment analysis of genes only deregulated by Put compared to flg22 and (Put+flg22) treatments.

Fig. S22. Effect of thermospermine and Spd on flg22-elicited ROS burst in the wild type.

Table S1.1. List of 554 differentially expressed genes at 24 h of Put treatment.

Table S1.2. List of 368 differentially expressed genes at 24 h of Spm treatment.

Table S1.3. List of 396 genes deregulated only by Put.

Table S1.4. List of 210 genes deregulated only by Spm.

Table S1.5. List of 158 common genes differentially expressed in Spm and Put treatments.

Table S2.1. List of 1415 differentially expressed genes at 24 h of flg22 treatment.

Table S2.2. GO analysis of genes deregulated in Table S2.1.

Table S2.3. List of 462 flg22-only genes that show significant expression differences at 24 h of flg22 treatment.

Table S2.4. GO analysis of genes deregulated in Table S2.3.

Table S2.5. Pathway enrichment analysis of genes deregulated in Table S2.3.

Table S2.6. List of 1534 differentially expressed genes at 24 h of (Spm+flg22) treatment.

Table S2.7. GO analysis of genes deregulated in Table S2.6.

Table S2.8. List of 868 common genes differentially expressed in flg22 and (Spm+flg22) treatments.

Table S2.9. GO analysis of genes deregulated in Table S2.8.

Table S2.10. Pathway enrichment analysis of genes deregulated in Table S2.8.

Table S2.11. List of 543 (Spm+flg22)-only genes that show significant differences in expression at 24 h of (Spm+flg22) treatment.

Table S2.12. GO analysis of genes deregulated in Table S2.11.

Table S2.13. Pathway enrichment analysis of genes deregulated in Table S2.11.

Table S2.14. List of 220 (Spm-only) genes that show significant differences in expression at 24 h of Spm treatment.

Table S2.15. GO analysis of genes deregulated in Table S2.14.

Table S2.16. List of 25 common genes differentially expressed in flg22 and Spm treatments.

Table S2.17. List of 63 common genes differentially expressed in Spm and (Spm+flg22) treatments.

Table S2.18. List of 60 common genes differentially expressed in Spm, flg22, and (Spm+flg22) treatments.

Table S2.19. GO analysis of genes deregulated in Table S2.18.

Table S3.1. List of 259 flg22-only genes that show significant expression differences at 24 h of flg22 treatment.

Table S3.2. GO analysis of genes deregulated in Table S3.1.

Table S3.3. Pathway enrichment analysis of genes deregulated in Table S3.1.

Table S3.4. List of 1395 differentially expressed genes at 24 h of (Put+flg22) treatment.

Table S3.5. GO analysis of genes deregulated in Table S3.4.

Table S3.6. List of 902 common genes differentially expressed in flg22 and (Put+flg22) treatments.

Table S3.7. GO analysis of genes deregulated in Table S3.6.

Table S3.8. Pathway enrichment analysis of genes deregulated in Table S3.6.

Table S3.9. List of 300 (Put+flg22)-only genes that show significant differences in expression at 24 h of (Put+flg22) treatment.

Table S3.10. GO analysis of genes deregulated in Table S3.9.

Table S3.11. Pathway enrichment analysis of genes deregulated in Table S3.9.

Table S3.12. List of 270 (Put-only) genes that show significant differences in expression at 24 h of Put treatment.

Table S3.13. GO analysis of genes deregulated in Table S3.12.

Table S3.14. Pathway enrichment analysis of genes deregulated in Table S3.12.

Table S3.15. List of 91 common genes differentially expressed in Put and flg22 treatments.

Table S3.16. GO analysis of genes deregulated in Table S3.15.

Table S3.17. List of 163 common genes differentially expressed in Put, flg22, and (Put+flg22) treatments.

Table S3.18. GO analysis of genes deregulated in Table S3.17.

Table S3.19. List of 30 common genes differentially expressed in Put and (Put+flg22) treatments.

erac411_suppl_Supplementary_Figure_S1-S22

erac411_suppl_Supplementary_Table_S1

erac411_suppl_Supplementary_Table_S2

erac411_suppl_Supplementary_Table_S3

## Data Availability

RNA-seq data have been deposited to ArrayExpress (https://www.ebi.ac.uk/arrayexpress/) under accession number E-MTAB-11820. All other data supporting the findings of this study are available within the paper and within its supplementary materials published online.
