# Supplementary material for: Spermine inhibits PAMP-induced ROS and Ca2+ burst and reshapes the transcriptional landscape of PAMP-triggered immunity in Arabidopsis
Source: J Exp Bot. 2022 Oct 20;74(1):427–42. doi: 10.1093/jxb/erac411 (PMC9786854; doi:10.1093/jxb/erac411)
Supplement: erac411_suppl_Supplementary_Figure_S1-S22 [file erac411_suppl_supplementary_figure_s1-s22.pdf]

SUPPLEMENTARY DATA

Spermine inhibits PAMP-induced ROS and Ca^{2+} burst and reshapes the transcriptional landscape of PTI in Arabidopsis

Chi Zhang, Kostadin E. Atanasov and Rubén Alcázar*

Department of Biology, Healthcare and Environment. Section of Plant Physiology, Faculty of Pharmacy and Food Sciences, Universitat de Barcelona, Av. Joan XXIII 27-31, 08028 Barcelona, Spain.

*Author for correspondence: ralcazar@ub.edu

SUPPLEMENTARY FIGURES S1 – S22

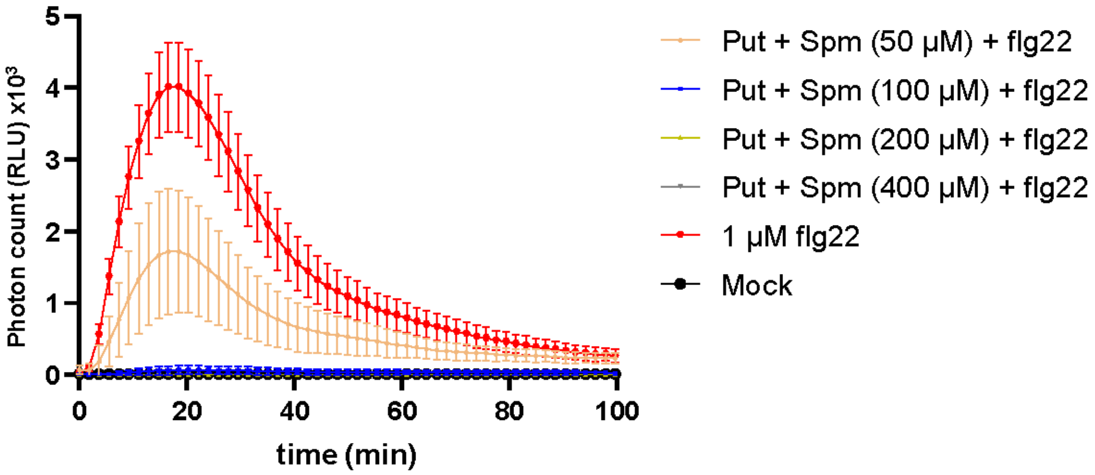

Figure S1. Effect of the Put and Spm cotreatment on flg22-elicited ROS burst. Leaf discs from 5-week-old wild-type plants were treated with flg22 (1 μM), and Put or Spm (50 μM to 400 μM). Values represent the mean ± S.E. from at least twelve replicates per treatment and are expressed in photon counts (relative light units, RLU).

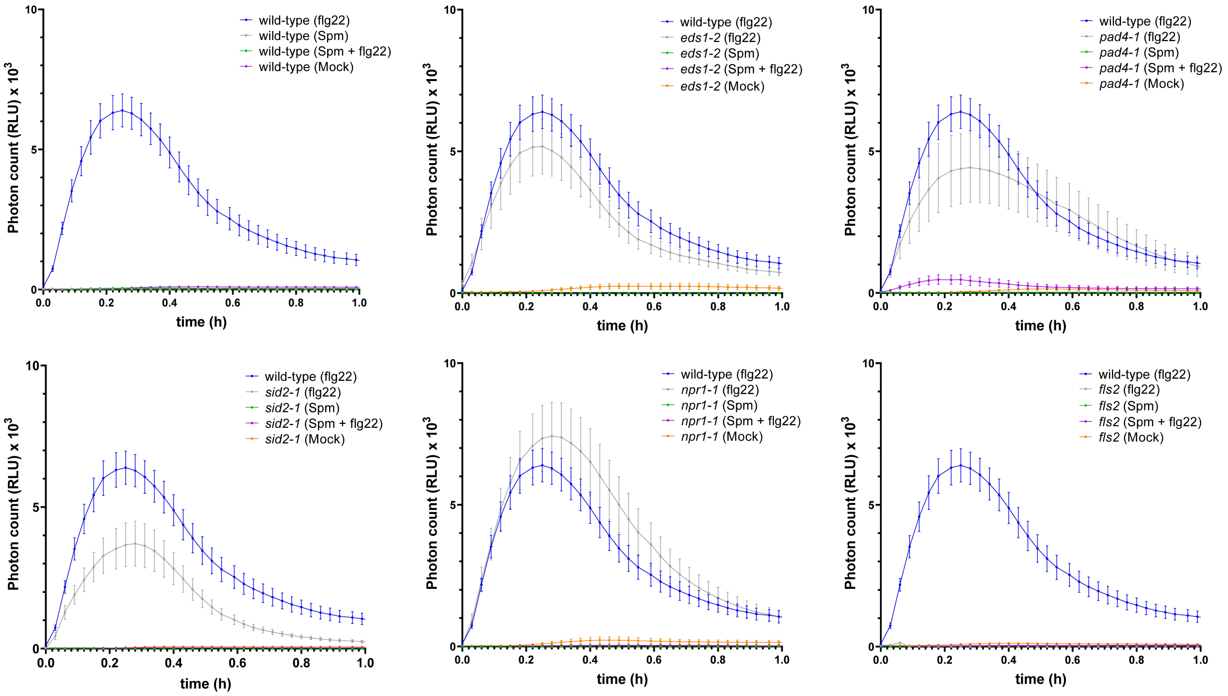

Figure S2. Effect of Spm on flg22-elicited ROS burst in *eds1-2*, *pad4-1*, *sid2-1*, *npr1-1* and *fls2* (negative control) mutants. Leaf discs from 5-week-old plants were treated with flg22 (1 µM), Spm (100 µM), Spm (100 µM) + flg22 (1 µM) or mock (water). Values represent the mean ± S.E. from at least twelve replicates per treatment and are expressed in photon counts (relative light units, RLU).

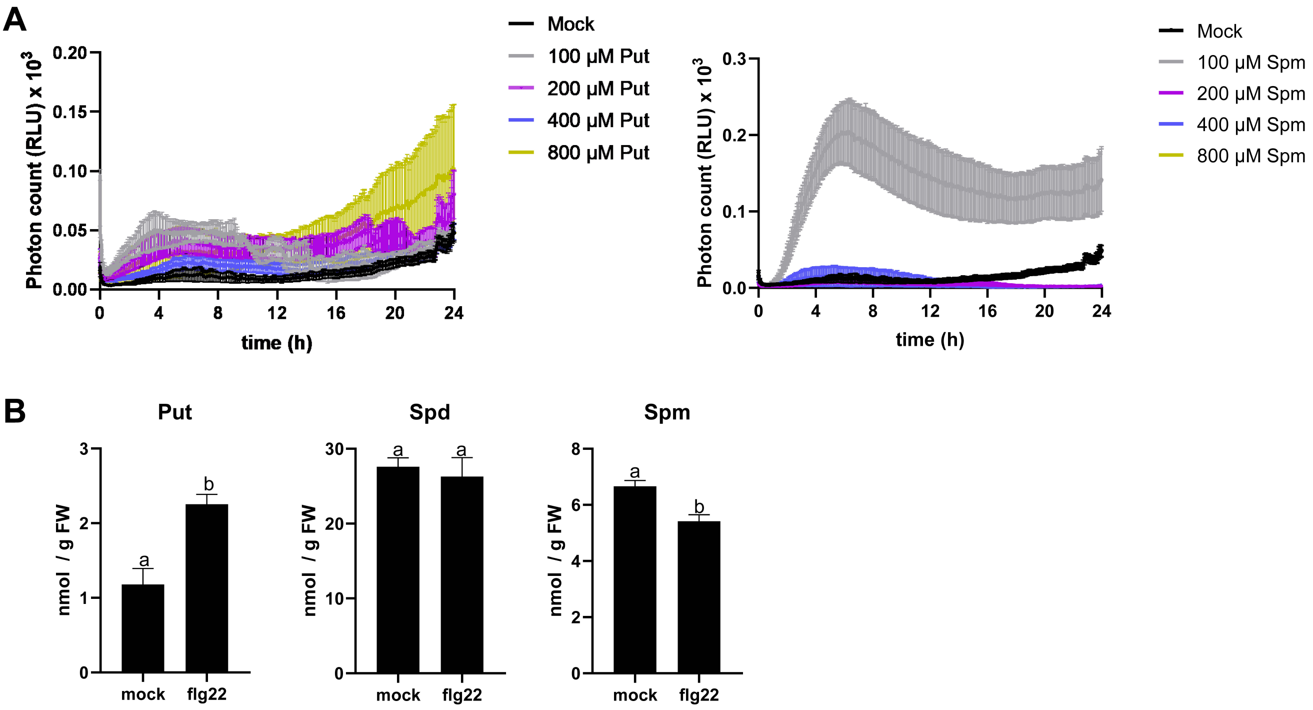

Figure S3. **(A)** ROS produced by Put and Spm treatments. Leaf discs from 5-week-old wild-type plants were incubated with different concentrations (100 µM to 800 µM) of Put, Spm and mock (water). Values represent the mean ± S.E. from at least twelve replicates per treatment and are expressed in photon counts (relative light units, RLU). **(B)** Free Put, Spd and Spm levels in wild-type plants at 24 h of treatment with flg22 (1 µM) or mock (water). Values represent the mean ± S.D. from three biological replicates per treatment. Letters indicate values that are significantly different according to Tukey's HSD test at $P < 0.05$.

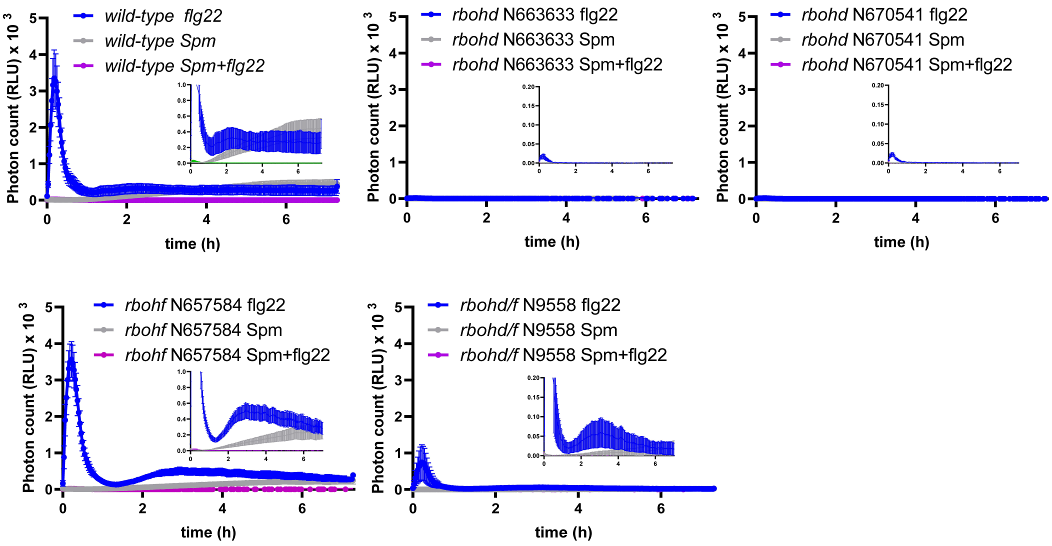

Figure S4. Effect of Spm on flg22-elicited ROS burst in *rbohd* (N663633 and N670541), *rbohf* (N657584) and double *rbohd/f* (N9558) mutants. Leaf discs from 5-week-old wild-type plants and mutants were treated with flg22 (1 µM), Spm (100 µM) or Spm (100 µM) + flg22 (1 µM). Values represent the mean ± S.E. from at least twelve replicates per treatment and are expressed in photon counts (relative light units, RLU).

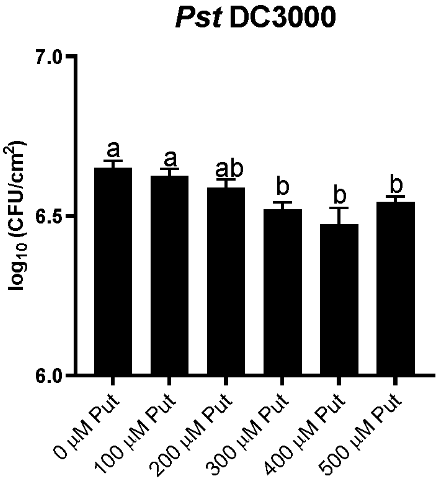

Figure S5. Analysis of *Pst* DC3000 disease resistance phenotypes in wild-type plants locally pretreated with different concentrations of Put (0 µM to 500 µM). Treatments were performed 24 h before *Pst* DC3000 infiltration ($OD_{600\ nm}$= 0.005). Bacterial numbers were assessed at 72 h post-inoculation and expressed as colony forming units (CFU) per cm^2 leaf area. Values are the mean from at least eight biological replicates ± SD. Letters indicate values that are significantly different according to Tukey's HSD test at $P < 0.05$.

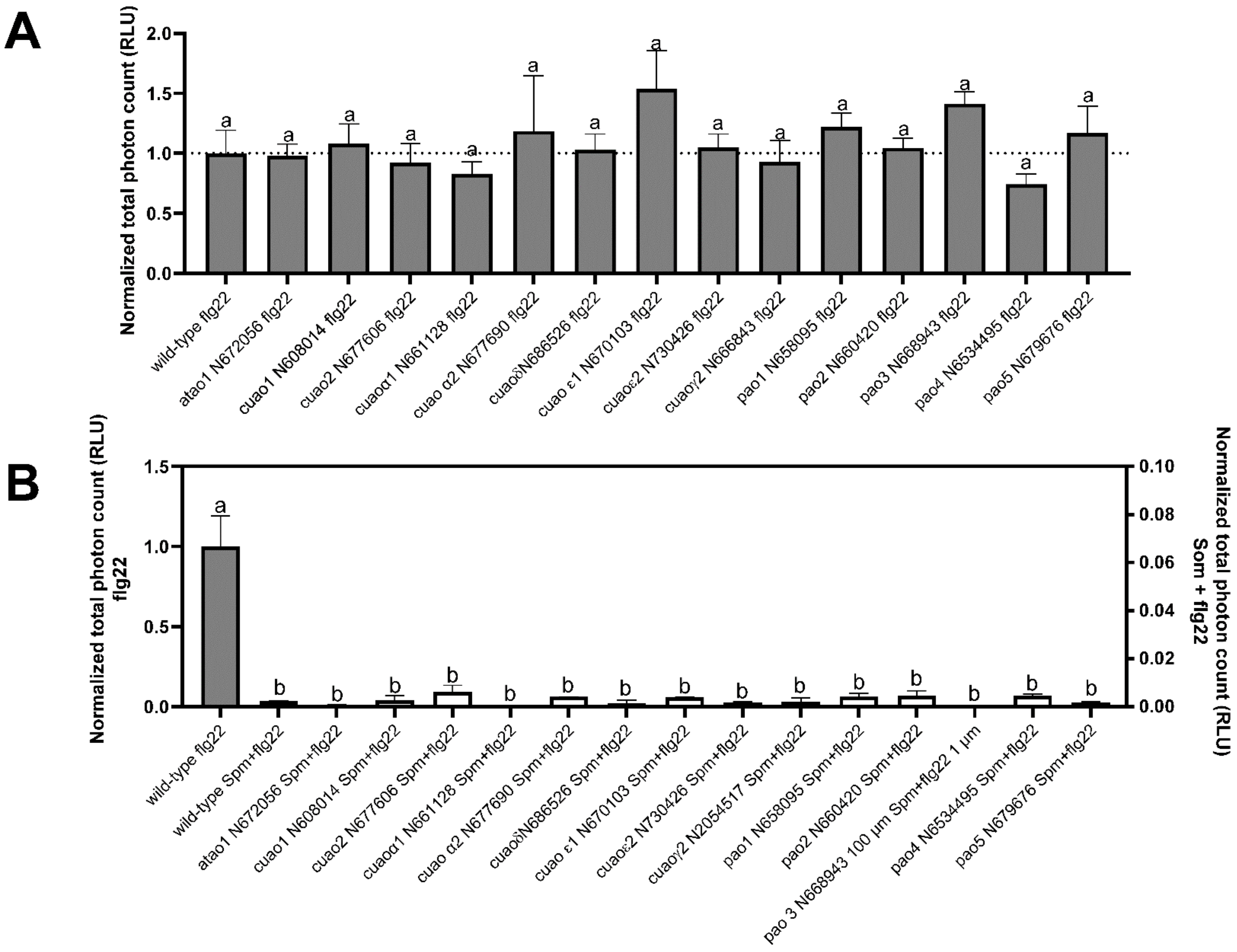

Figure S6. (A) Flg22-elicited ROS and **(B)** effect of Spm on flg22-elicited ROS production in *CuAO* mutants (*atao1*, *cuao1*, *cuao2*, *cuaoa1*, *cuao*a2, *cuao*d, *cuao*e1, *cuao*e2, *cuao*g2) and *PAO* mutants (*pao1*, *pao2*, *pao3*, *pao4* and *pao5*) in comparison to the wild-type. The total sum of RLU (total photon counts) in each genotype was normalized to the total photon counts in the wild-type reference. Values represent the mean ± S.E. of the normalized values from at least twelve replicates per treatment. Letters indicate values that are significantly different according to Tukey's HSD test at $P < 0.05$.

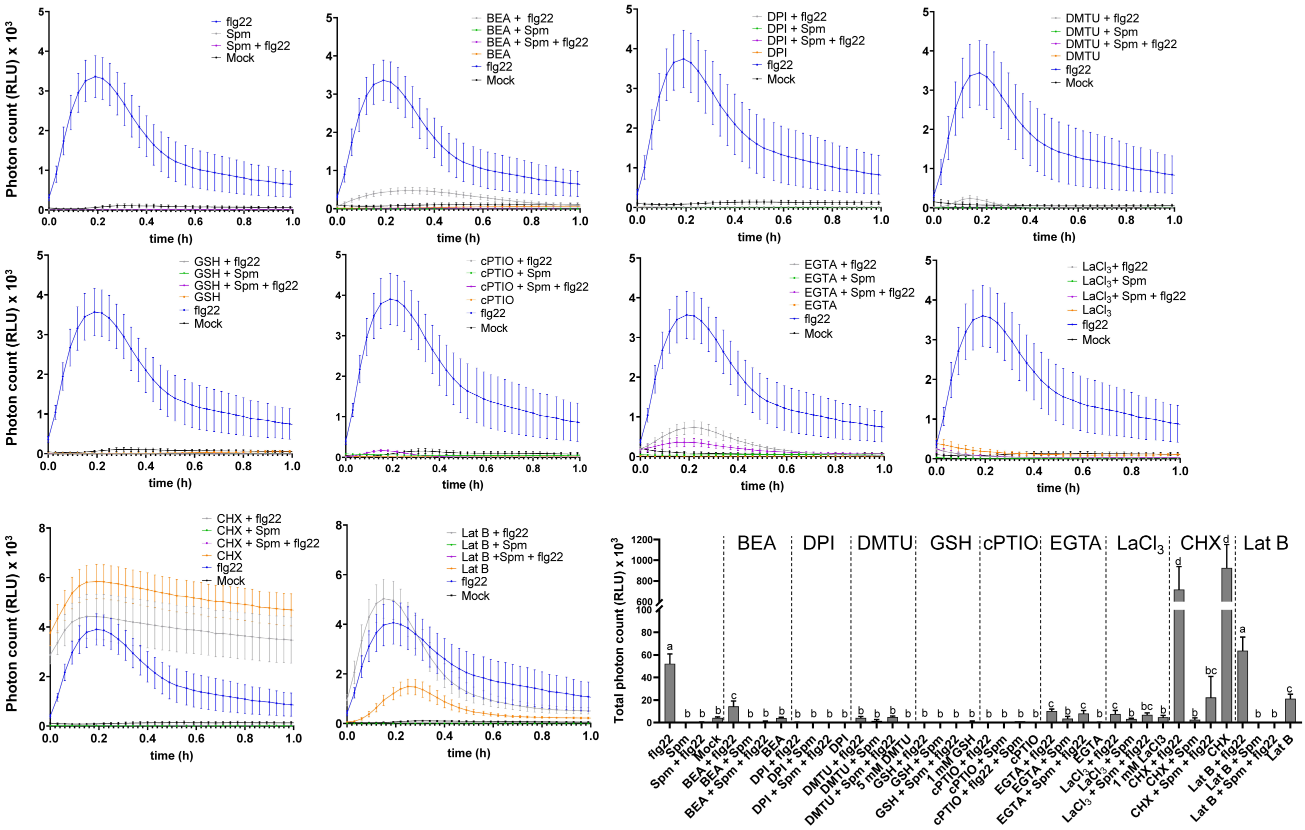

Figure S7. Effect of 2-bromoethylamine BEA (5 mM), diphenyleneiodonium chloride (DPI, 20 µM), dimethylthiourea (DMTU, 5 mM), reduced L-glutathione (GSH, 1 mM), carboxy-PTIO (cPTIO, 100 µM), EGTA (2 mM), $LaCl_3$ (1 mM), cycloheximide (CHX, 300 µM) and Latrunculin B (Lat B, 20 µM) on Spm inhibition of flg22-triggered ROS burst in wild-type plants. Leaf discs from 5-week-old plants were pretreated with the different chemicals 3 h before Spm (100 µM), flg22 (1 µM) and Spm (100 µM) + flg22 (1 µM) elicitation. Photon counts (relative light units, RLU) were determined over time. Values represent the mean ± S.E. from at least twelve replicates per treatment. Letters indicate values that are significantly different according to Tukey's HSD test at $P < 0.05$.

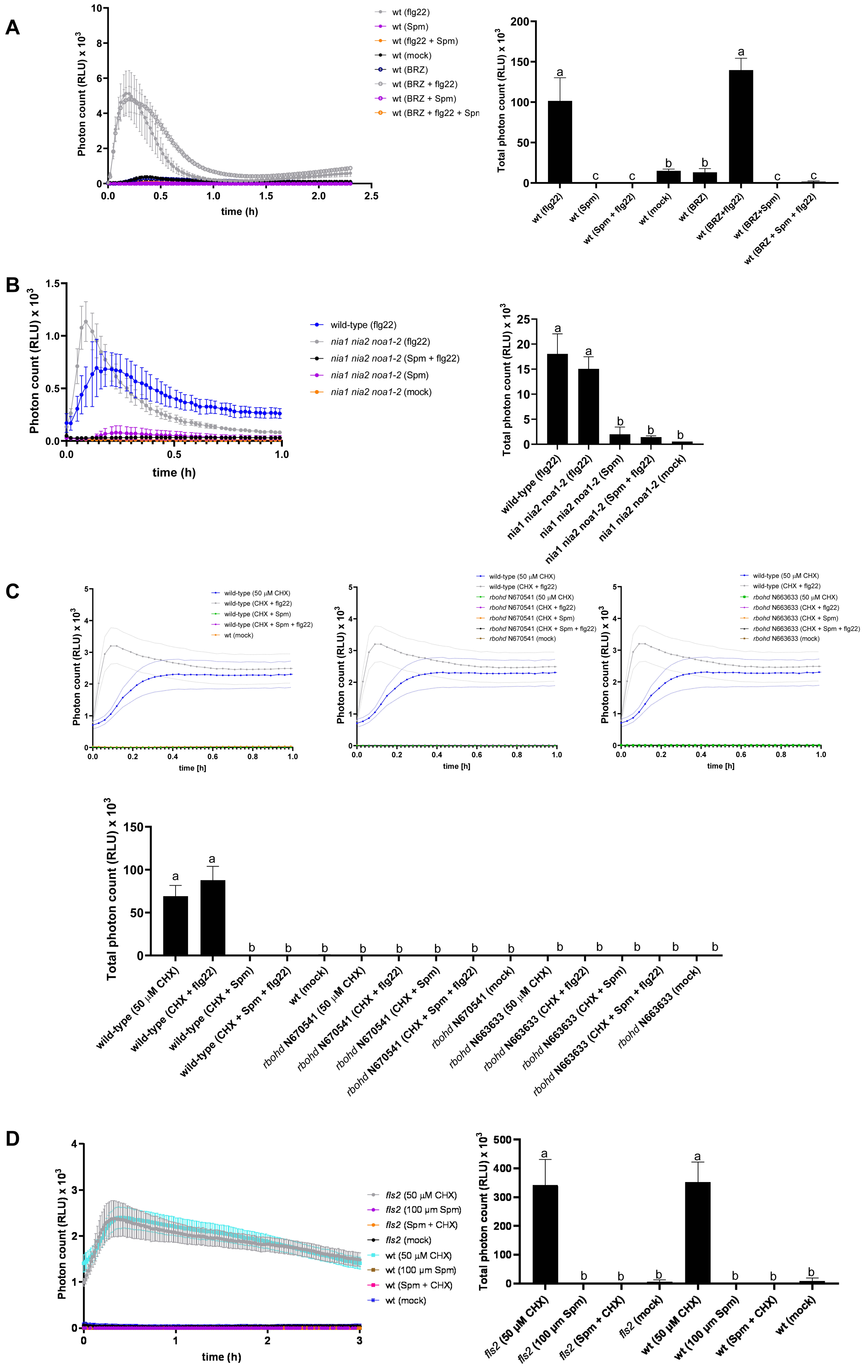
A
Photon count (RLU) x 10^3
time (h)
wt (flg22)
wt (Spm)
wt (flg22 + Spm)
wt (mock)
wt (BRZ)
wt (BRZ + flg22)
wt (BRZ + Spm)
wt (BRZ + flg22 + Spm
Total photon count (RLU) x 10^3
wt (flg22)
wt (Spm)
wt (Spm + flg22)
wt (mock)
wt (BRZ)
wt (BRZ+flg22)
wt (BRZ+Spm)
wt (BRZ + Spm + flg22)
B
Photon count (RLU) x 10^3
time (h)
wild-type (flg22)
nia1 nia2 noa1-2 (flg22)
nia1 nia2 noa1-2 (Spm + flg22)
nia1 nia2 noa1-2 (Spm)
nia1 nia2 noa1-2 (mock)
Total photon count (RLU) x 10^3
C
Photon count (RLU) x 10^3
time [h]
wild-type (50 µM CHX)
wild-type (CHX + flg22)
wild-type (CHX + Spm)
wild-type (CHX + Spm + flg22)
wt (mock)
rbohd N670541 (50 µM CHX)
rbohd N670541 (CHX + flg22)
rbohd N670541 (CHX + Spm)
rbohd N670541 (CHX + Spm + flg22)
rbohd N670541 (mock)
rbohd N663633 (50 µM CHX)
rbohd N663633 (CHX + flg22)
rbohd N663633 (CHX + Spm)
rbohd N663633 (CHX + Spm + flg22)
rbohd N663633 (mock)
Total photon count (RLU) x 10^3
D
Photon count (RLU) x 10^3
time (h)
fls2 (50 µM CHX)
fls2 (100 µm Spm)
fls2 (Spm + CHX)
fls2 (mock)
wt (50 µM CHX)
wt (100 µm Spm)
wt (Spm + CHX)
wt (mock)
Total photon count (RLU) x 10^3

Figure S8. (A) Effect of brassinazole (BRZ, 2.5 µM) on Spm inhibition of flg22-triggered ROS burst in the wild-type. **(B)** Effect of Spm on flg22-elicited ROS burst in 3-week-old *nia1 nia2 noa1-2* triple mutant. **(C,D)** Effect of cycloheximide (CHX, 50 µM) on Spm inhibition of flg22-triggered ROS burst in **(B)** wild-type and *rbohd* **(C)** wild-type (wt) and *fls2*. Pharmacological treatments were performed as described in Figure S7. Values represent the mean ± S.E. from at least twelve replicates per treatment and are expressed in photon counts (relative light units, RLU). Letters indicate values that are significantly different according to Tukey's HSD test at $P < 0.05$.

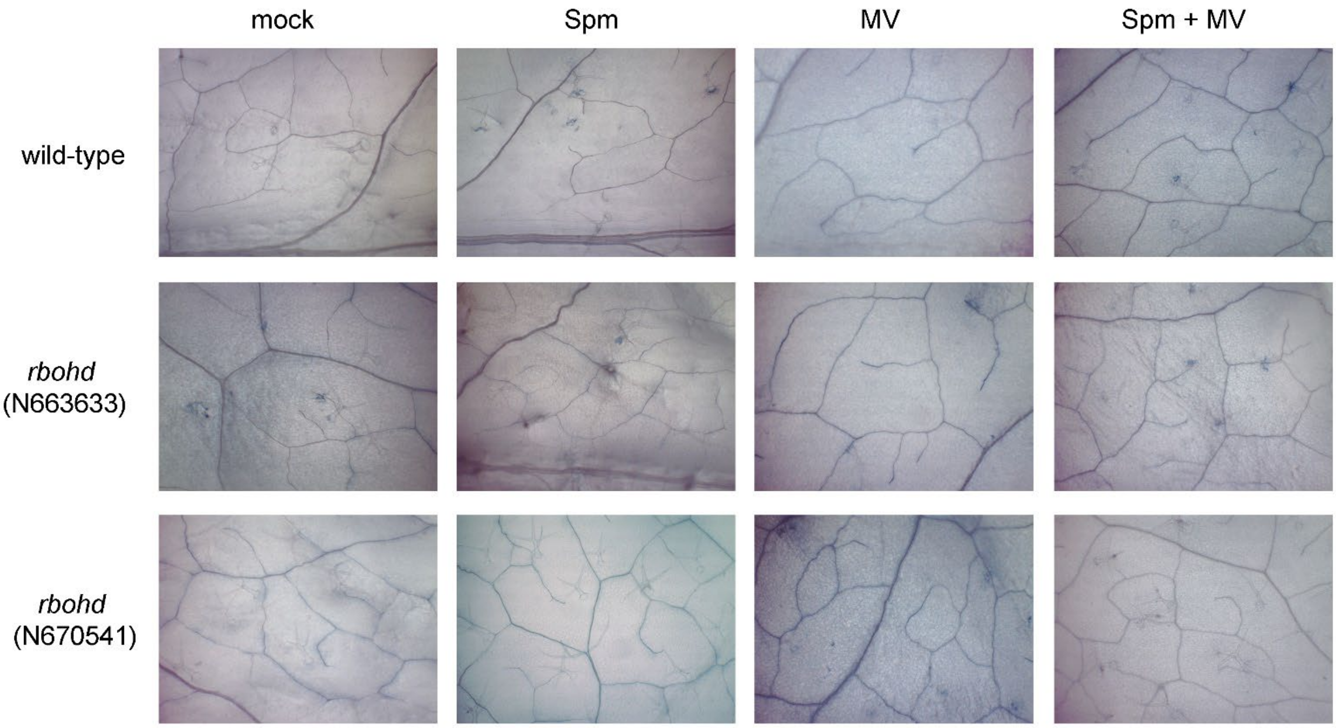

Figure S9. Trypan blue staining of wild-type and *rbohd* leaves infiltrated with Spm (100 µM), methyl viologen (MV, 100 µM) or both (100 µM Spm + 100 µM MV). Staining was performed at 24 h of treatment.

A

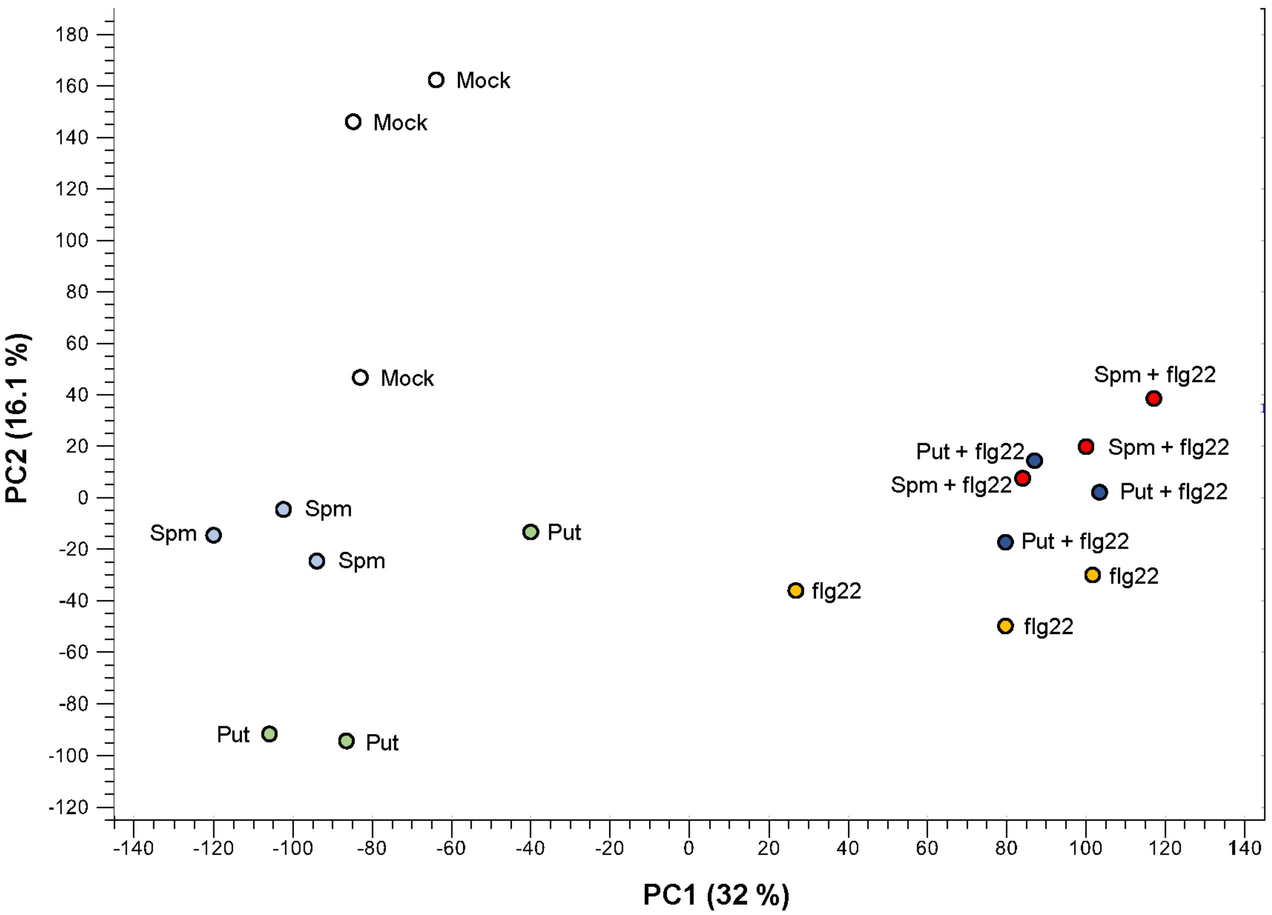
PC2 (16.1 %)
PC1 (32 %)
Mock
Mock
Mock
Spm
Spm
Spm
Put
Put
Put
flg22
flg22
flg22
Spm + flg22
Spm + flg22
Spm + flg22
Put + flg22
Put + flg22
Put + flg22

B

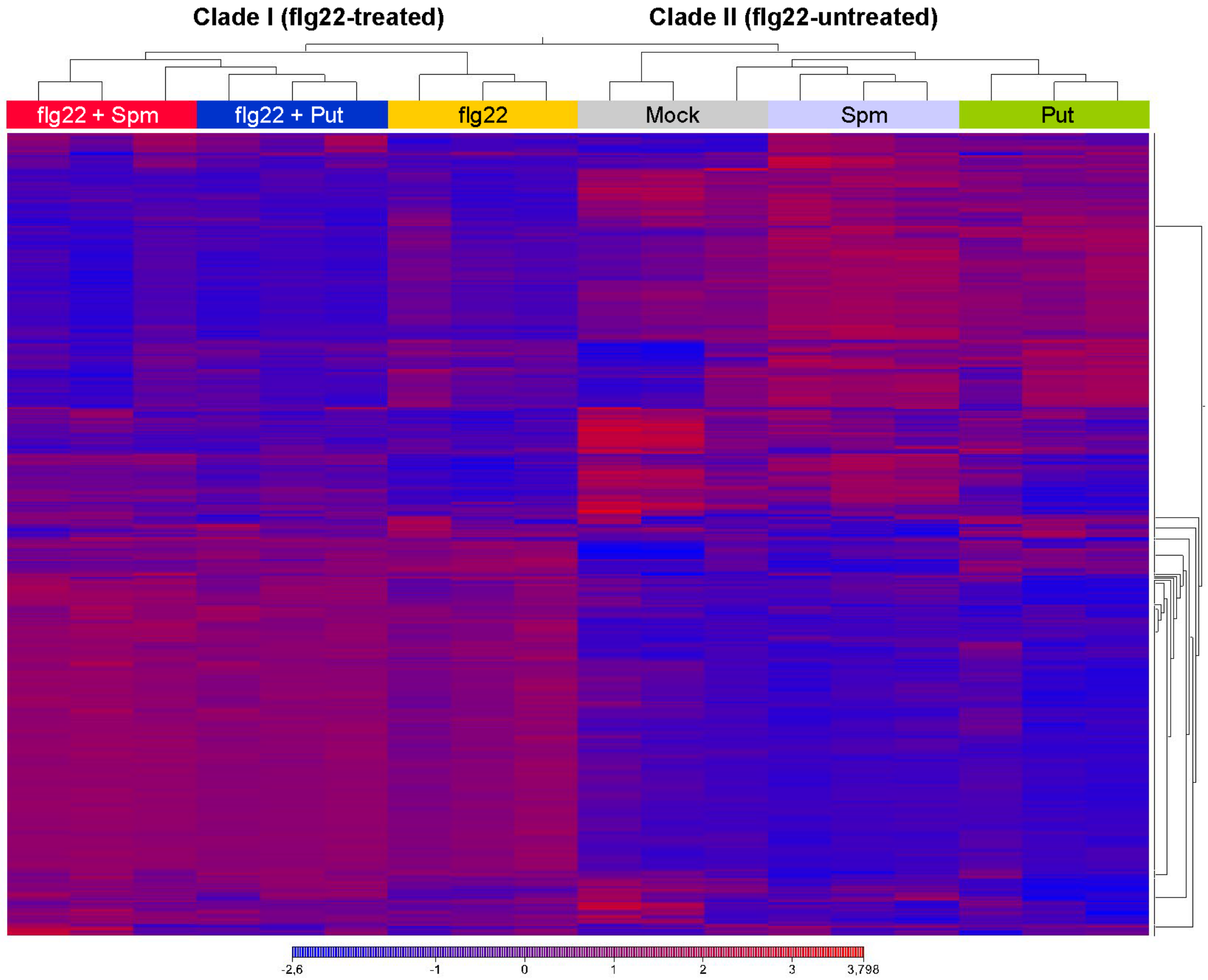
Clade I (flg22-treated)
Clade II (flg22-untreated)
flg22 + Spm
flg22 + Put
flg22
Mock
Spm
Put
-2,6
-1
0
1
2
3
3,798

Figure S10. (A) Principal component analysis (PCA) and **(B)** Hierarchical clustering analysis (HCA) of RNA-seq gene expression data obtained from 5-week-old wild-type plants treated with Put (100 µM), Spm (100 µM), flg22 (1 µM), Put (100 µM) + flg22 (1 µM), Spm (100 µM) + flg22 (1 µM) and mock (water) for 24 h. Each treatment was performed in three biological replicates.

A

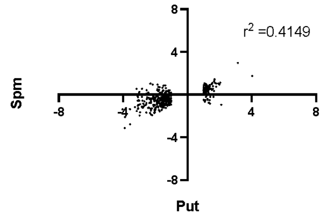

Put and Spm

Upregulated genes (83)			
**GO biological process**		**Fold**	**P-value**
cell wall biogenesis	GO:0042546	12.07	6.28E-03
plant-type cell wall organization or biogenesis	GO:0071669	10.99	1.14E-02
cell wall organization or biogenesis	GO:0071554	7.34	1.14E-02
cell growth	GO:0016049	8.76	1.16E-02
unidimensional cell growth	GO:0009826	10.3	1.73E-02
polysaccharide metabolic process	GO:0005976	8.01	2.20E-02
hemicellulose metabolic process	GO:0010410	18.69	2.56E-02
growth	GO:0040007	7.61	3.16E-02
xyloglucan metabolic process	GO:0010411	28.64	4.34E-02
developmental growth involved in morphogenesis	GO:0060560	8.85	4.52E-02
**Downregulated genes (71)**			
**GO biological process**		**Fold**	**P-value**
response to chemical	GO:0042221	5.33	9.89E-11
response to stimulus	GO:0050896	3.1	4.32E-08
response to oxygen-containing compound	GO:1901700	6.63	5.99E-08
response to organic substance	GO:0010033	6.09	6.77E-08
response to hormone	GO:0009725	6.88	5.15E-07
response to water deprivation	GO:0009414	14.97	5.66E-07
response to endogenous stimulus	GO:0009719	6.72	7.42E-07
regulation of biological process	GO:0050789	2.96	1.06E-06
response to acid chemical	GO:0001101	13.46	1.68E-06
response to stress	GO:0006950	3.76	6.99E-06

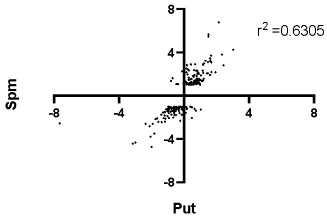

Put only

Upregulated genes (114)			
**GO biological process**		**Fold**	**P-value**
translation	GO:0006412	14.3	1.92E-17
gene expression	GO:0010467	5.67	8.12E-12
organic substance biosynthetic process	GO:1901576	4.03	4.60E-10
ribosome biogenesis	GO:0042254	11.43	4.91E-07
primary metabolic process	GO:0044238	2	5.01E-04
ribosome assembly	GO:0042255	25.71	5.83E-04
cytoplasmic translation	GO:0002181	23.44	9.76E-04
non-membrane-bounded organelle assembly	GO:0140694	12.43	3.25E-02
**Downregulated genes (282)**			
**GO biological process**		**Fold**	**P-value**
response to abiotic stimulus	GO:0009628	5.01	1.13E-29
response to stress	GO:0006950	3.84	5.38E-28
response to chemical	GO:0042221	4.03	2.94E-23
cellular response to decreased oxygen levels	GO:0036294	14.79	2.44E-18
response to oxygen-containing compound	GO:1901700	4.28	5.13E-13
cellular response to stimulus	GO:0051716	3.06	2.32E-11
response to organic substance	GO:0010033	3.57	1.18E-09
regulation of transcription, DNA-templated	GO:0006355	2.93	7.05E-08
response to external biotic stimulus	GO:0043207	3.82	6.90E-07
regulation of cellular metabolic process	GO:0031323	2.5	8.31E-07

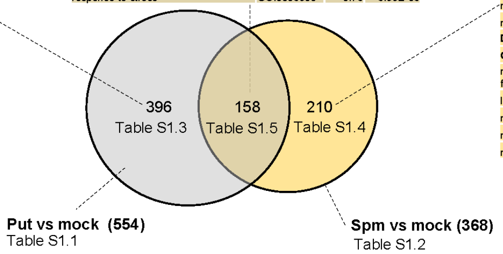

Spm only

Upregulated genes (106)			
**GO biological process**		**Fold**	**P-value**
response to hormone	GO:0009725	5.2	2.23E-05
response to endogenous stimulus	GO:0009719	5.08	3.21E-05
response to chemical	GO:0042221	3.52	3.77E-05
response to organic substance	GO:0010033	4.13	2.96E-04
response to oxygen-containing compound	GO:1901700	3.99	5.80E-03
response to lipid	GO:0033993	5.02	3.52E-02
response to cytokinin	GO:0009735	17.15	3.99E-02
**Downregulated genes (104)**			
**GO biological process**		**Fold**	**P-value**
regulation of DNA-binding transcription factor activity	GO:0051090	92.78	2.37E-02
leaf senescence	GO:0010150	16.87	4.26E-02
response to other organism	GO:0051707	4.38	2.21E-02
response to biotic stimulus	GO:0009607	4.37	2.25E-02
response to stimulus	GO:0050896	2.31	1.39E-03

B

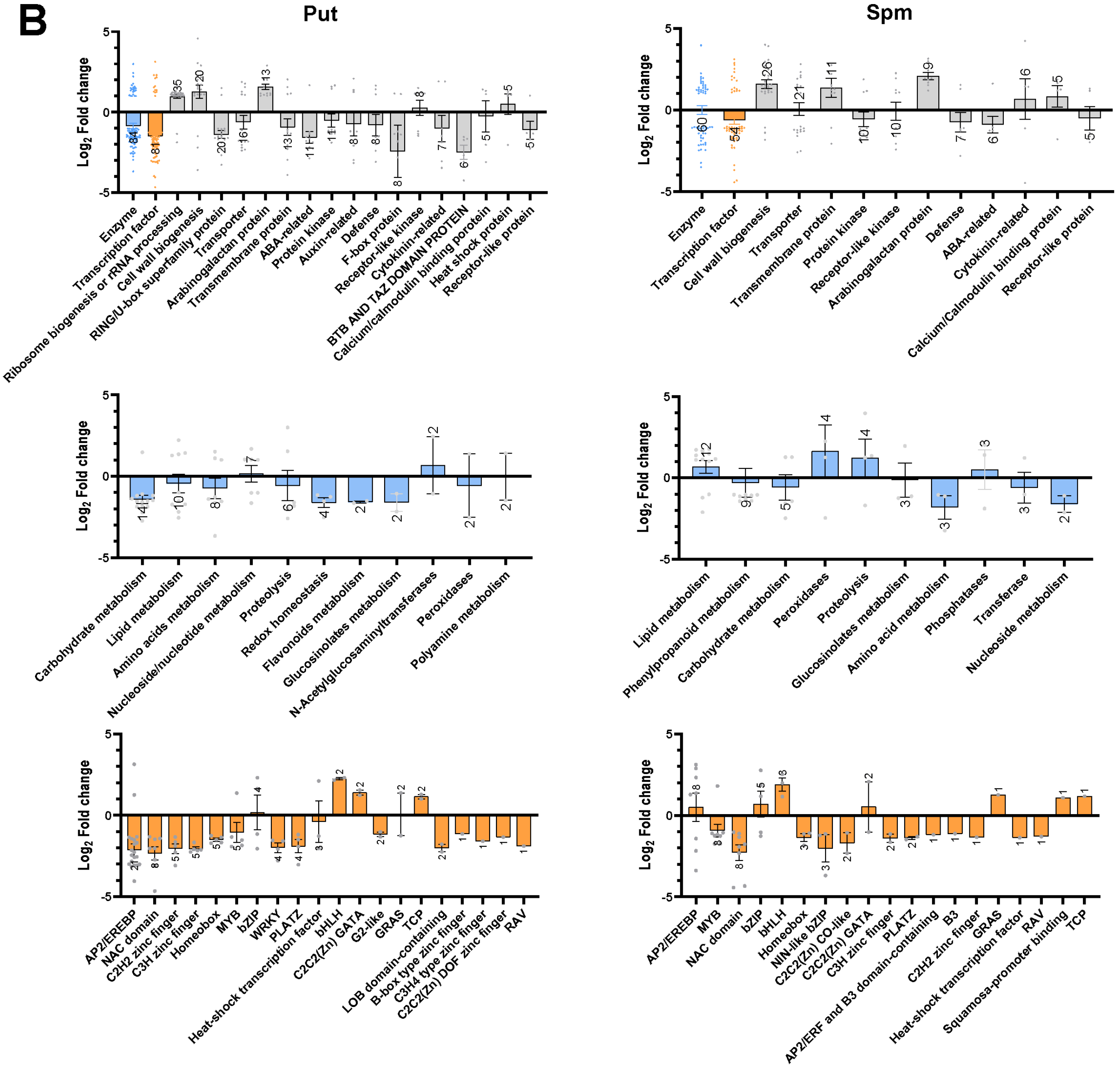

Figure S11. (A) Venn diagram, gene ontology (GO) and expression correlation analyses of genes significantly deregulated (fold-change ≥ 2; Bonferroni corrected *P*-value ≤ 0.05) in response to Put (100 μM) and Spm (100 μM) at 24 h of treatment in the wild-type. **(B)** Molecular functions, main enzymatic activities and TF families of genes differentially expressed in Put and Spm treatments. Bars indicate the mean expression ± S.E. The number of genes within each category are indicated on top of the bar and listed in Tables S1.1 to S1.5.

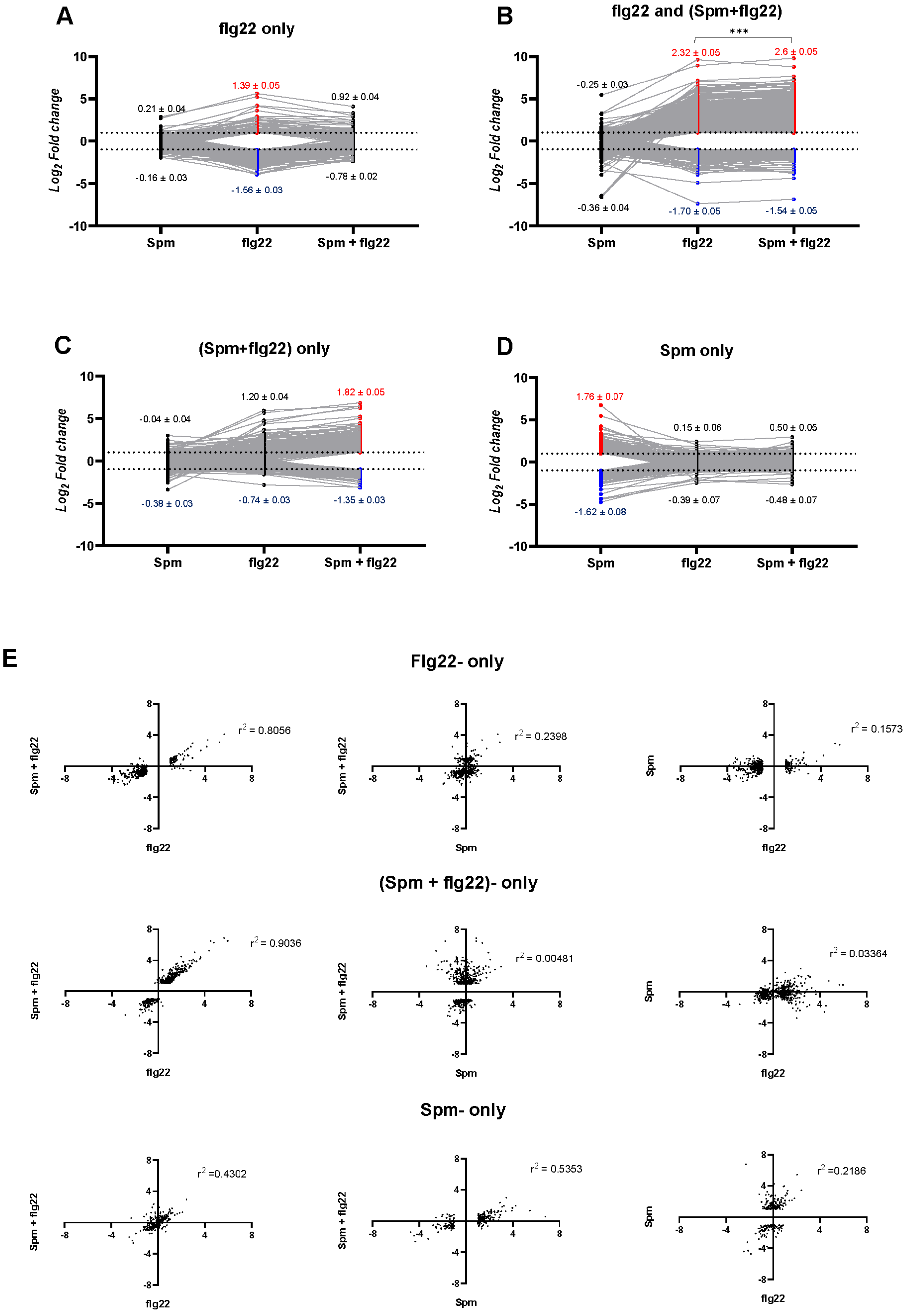
A
flg22 only
Log2 Fold change
0.21 ± 0.04
1.39 ± 0.05
0.92 ± 0.04
-0.16 ± 0.03
-1.56 ± 0.03
-0.78 ± 0.02
Spm
flg22
Spm + flg22
B
flg22 and (Spm+flg22)
***
-0.25 ± 0.03
2.32 ± 0.05
2.6 ± 0.05
-0.36 ± 0.04
-1.70 ± 0.05
-1.54 ± 0.05
C
(Spm+flg22) only
-0.04 ± 0.04
1.20 ± 0.04
1.82 ± 0.05
-0.38 ± 0.03
-0.74 ± 0.03
-1.35 ± 0.03
D
Spm only
1.76 ± 0.07
0.15 ± 0.06
0.50 ± 0.05
-1.62 ± 0.08
-0.39 ± 0.07
-0.48 ± 0.07
E
Flg22- only
r2 = 0.8056
r2 = 0.2398
r2 = 0.1573
(Spm + flg22)- only
r2 = 0.9036
r2 = 0.00481
r2 = 0.03364
Spm- only
r2 =0.4302
r2 = 0.5353
r2 =0.2186
Spm + flg22
Spm
flg22

Figure S12. Mean expression values and correlation analyses of wild-type (Col-0) plants treated with flg22 (1 µM), Spm (100 µM) and Spm (100 µM) + flg22 (1 µM). **(A)** Genes only significantly deregulated by flg22 treatment. **(B)** Common genes deregulated by flg22 and (Spm + flg22) treatments. **(C)** Genes only deregulated by (Spm + flg22) treatment. **(D)** Genes only deregulated by Spm treatment. Expression values (Log2) are relative to the mock (H_2O). The mean expression ± S.E of upregulated and downregulated genes is shown for each treatment. Asterisks indicate significant differences according to Wilcoxon signed-rank test (***p<0.001). **(E)** Expression correlation between flg22, Spm and (Spm + flg22) treatments.

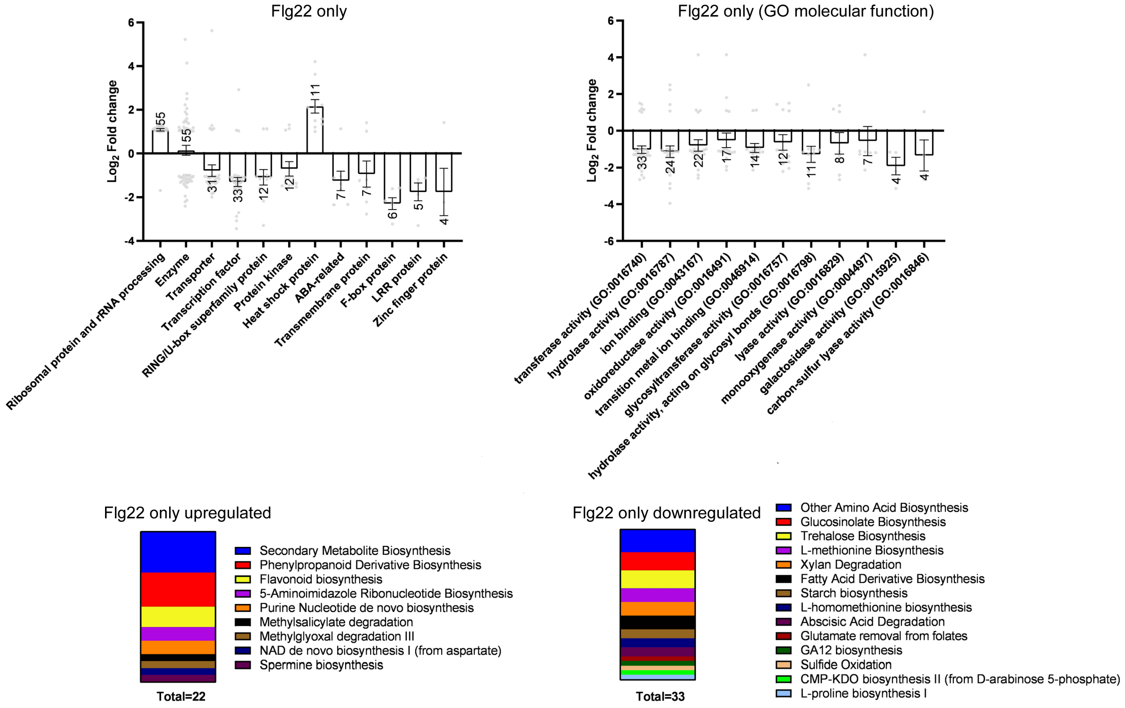

Figure S13. Molecular function categorization and metabolic pathway enrichment analysis of genes only deregulated by flg22 compared to Spm and (Spm + flg22) treatments in the wild-type. Bars indicate the mean expression ± S.E in each category.

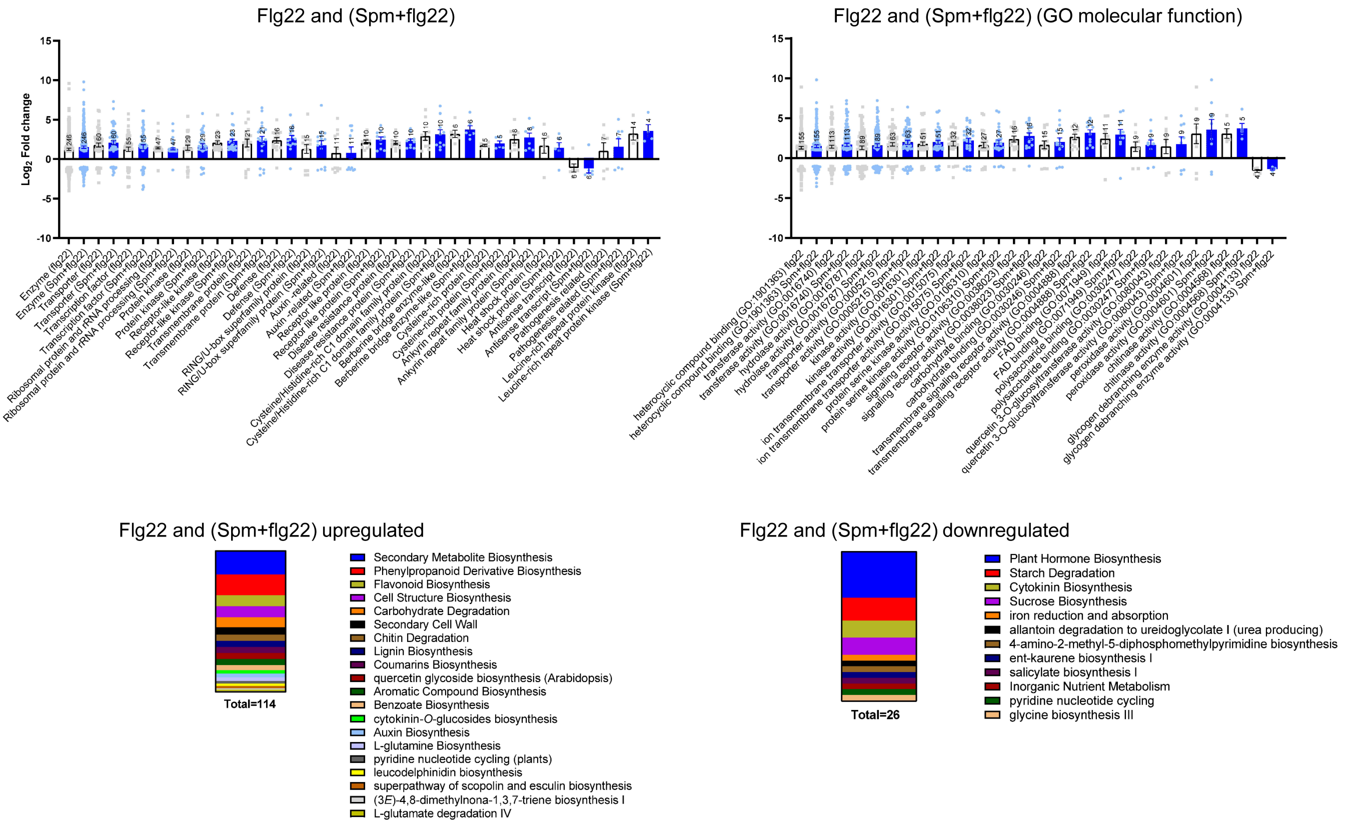

Figure S14. Molecular function categorization and metabolic pathway enrichment analysis of genes commonly deregulated in flg22 and (Spm + flg22) treatments in the wild-type. Bars indicate the mean expression ± S.E in each category.

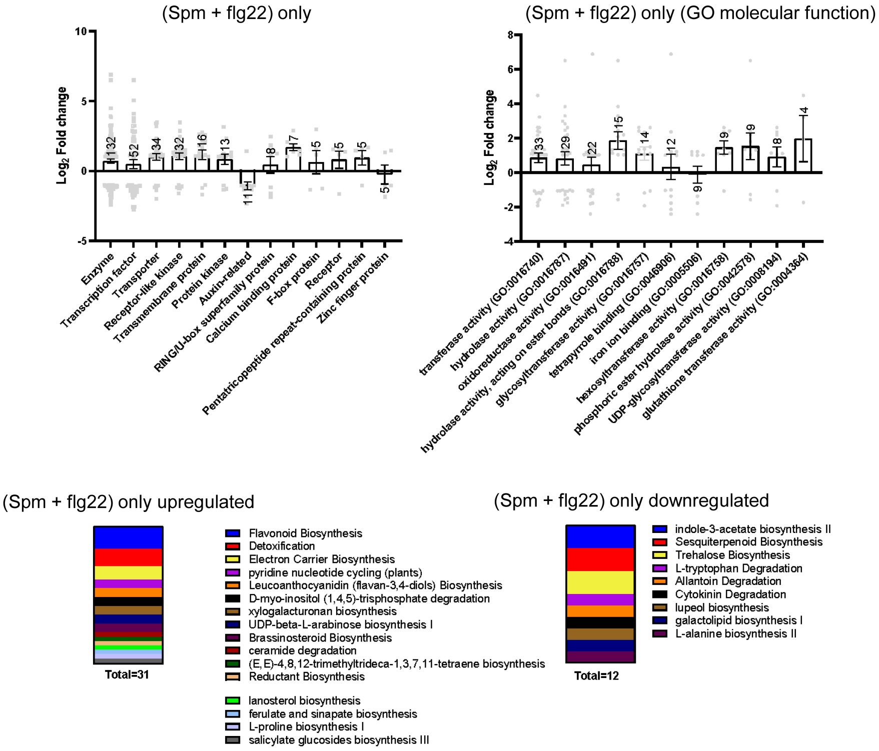

Figure S15. Molecular function categorization and metabolic pathway enrichment analysis of genes only differentially expressed in (Spm + flg22) compared to flg22 and Spm treatments. Bars indicate the mean expression ± S.E in each category.

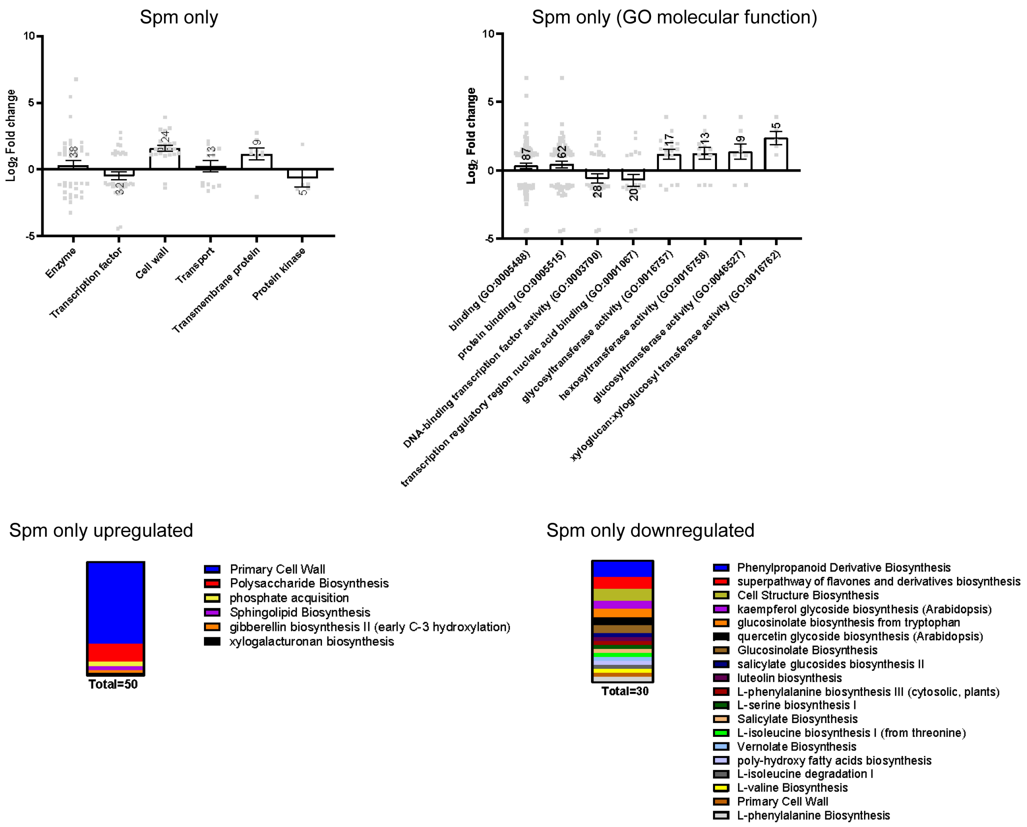

Figure S16. Molecular function categorization and metabolic pathway enrichment analysis of genes only differentially expressed in Spm treatment compared to flg22 and (Spm + flg22) treatments. Bars indicate the mean expression ± S.E in each category.

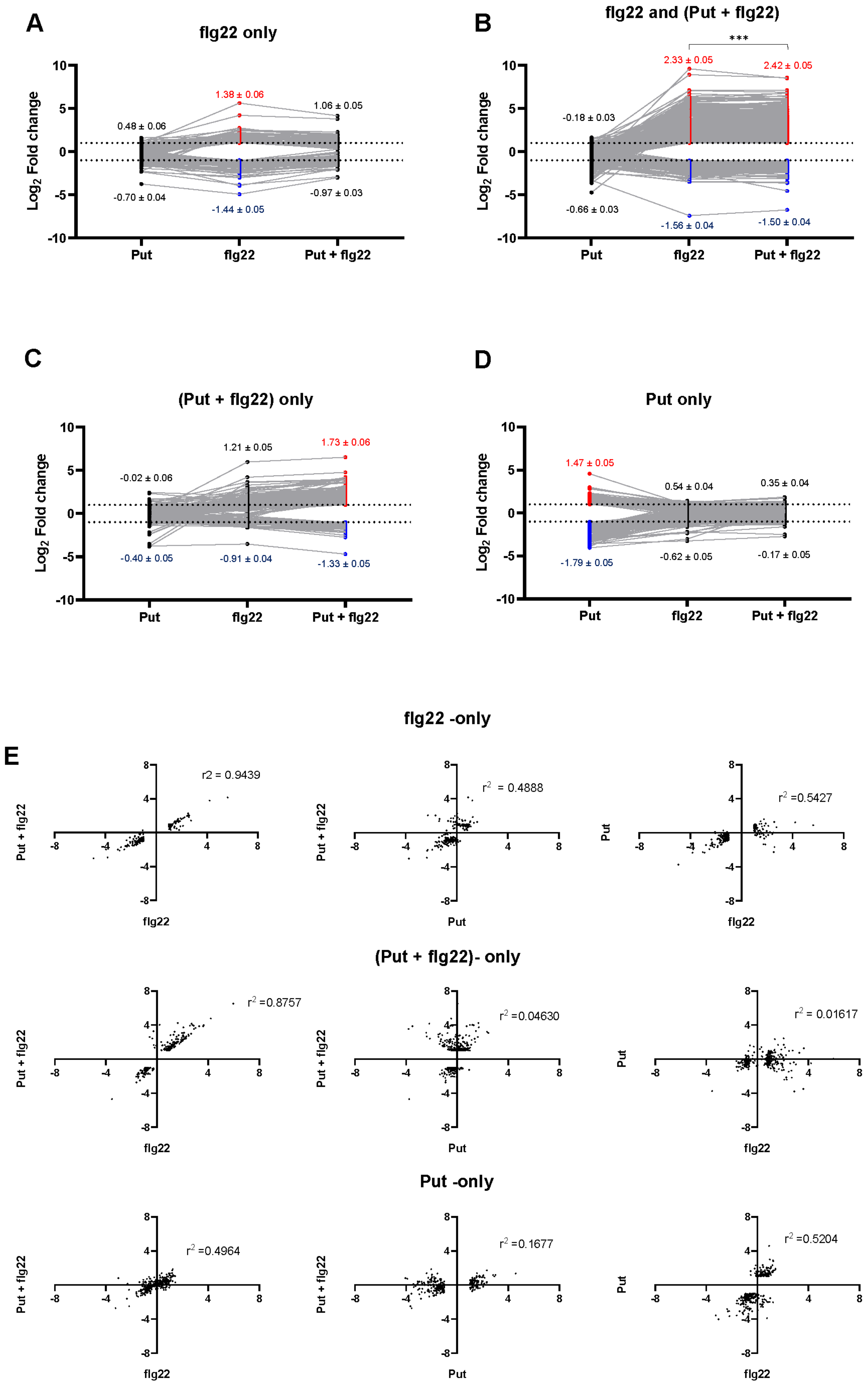
A
flg22 only
Log2 Fold change
0.48 ± 0.06
1.38 ± 0.06
1.06 ± 0.05
-0.70 ± 0.04
-1.44 ± 0.05
-0.97 ± 0.03
Put
flg22
Put + flg22
B
flg22 and (Put + flg22)
***
2.33 ± 0.05
2.42 ± 0.05
-0.18 ± 0.03
-0.66 ± 0.03
-1.56 ± 0.04
-1.50 ± 0.04
C
(Put + flg22) only
1.21 ± 0.05
1.73 ± 0.06
-0.02 ± 0.06
-0.40 ± 0.05
-0.91 ± 0.04
-1.33 ± 0.05
D
Put only
1.47 ± 0.05
0.54 ± 0.04
0.35 ± 0.04
-1.79 ± 0.05
-0.62 ± 0.05
-0.17 ± 0.05
flg22 -only
E
r2 = 0.9439
r2 = 0.4888
r2 =0.5427
(Put + flg22)- only
r2 =0.8757
r2 =0.04630
r2 = 0.01617
Put -only
r2 =0.4964
r2 =0.1677
r2 =0.5204
Put + flg22
Put
flg22

Figure S17. Mean expression values and correlation analyses of wild-type (Col-0) plants treated with flg22 (1 µM), Put (100 µM) and Put (100 µM) + flg22 (1 µM). **(A)** Genes only significantly deregulated by flg22 treatment. **(B)** Common genes deregulated by flg22 and (Put + flg22) treatments. **(C)** Genes only deregulated by (Put + flg22) treatment. **(D)** Genes only deregulated by Put treatment. Expression values (Log2) are relative to the mock (H_2O). The mean expression ± S.E of upregulated and downregulated genes is shown for each treatment. Asterisks indicate significant differences according to Wilcoxon signed-rank test (***p<0.001). **(E)** Expression correlation between flg22, Put and (Put + flg22) treatments.

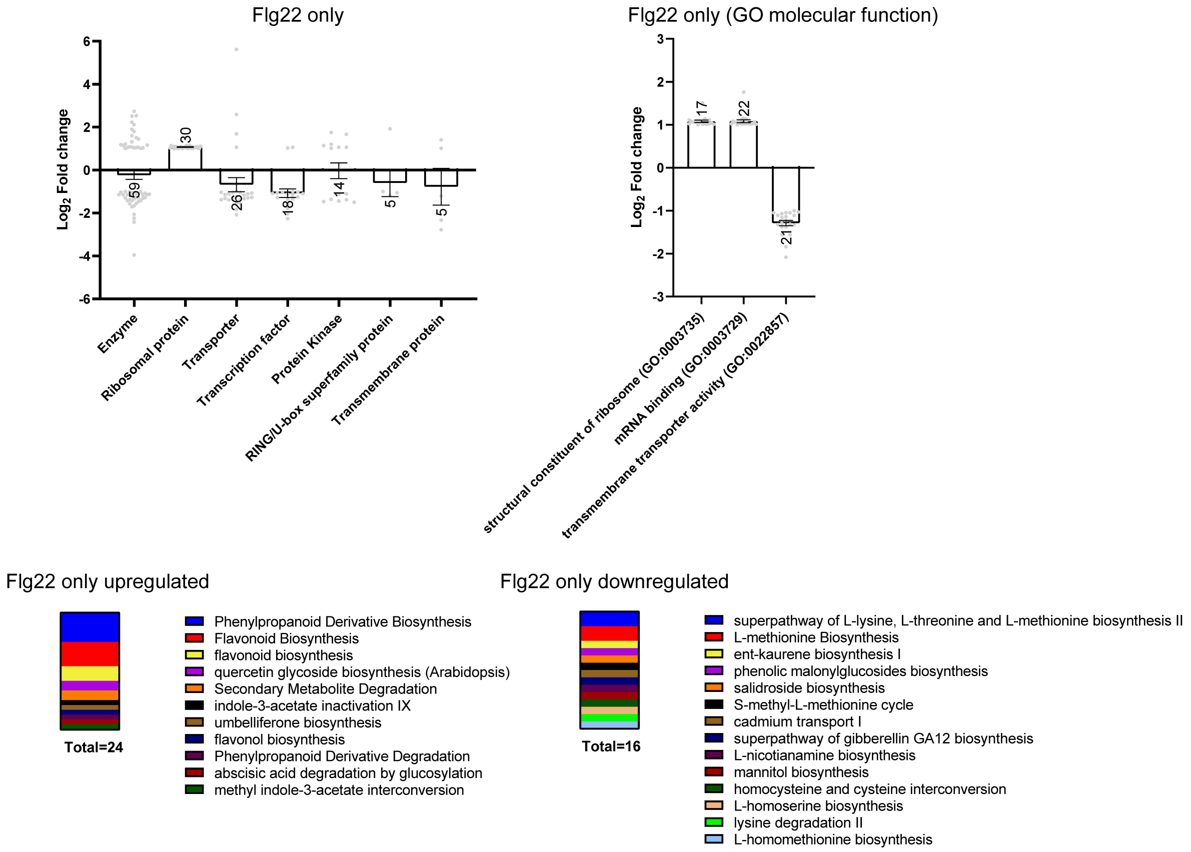

Figure S18. Molecular function categorization and metabolic pathway enrichment analysis of genes only deregulated by flg22 compared to Put and (Put + flg22) treatments. Bars indicate the mean expression ± S.E in each category.

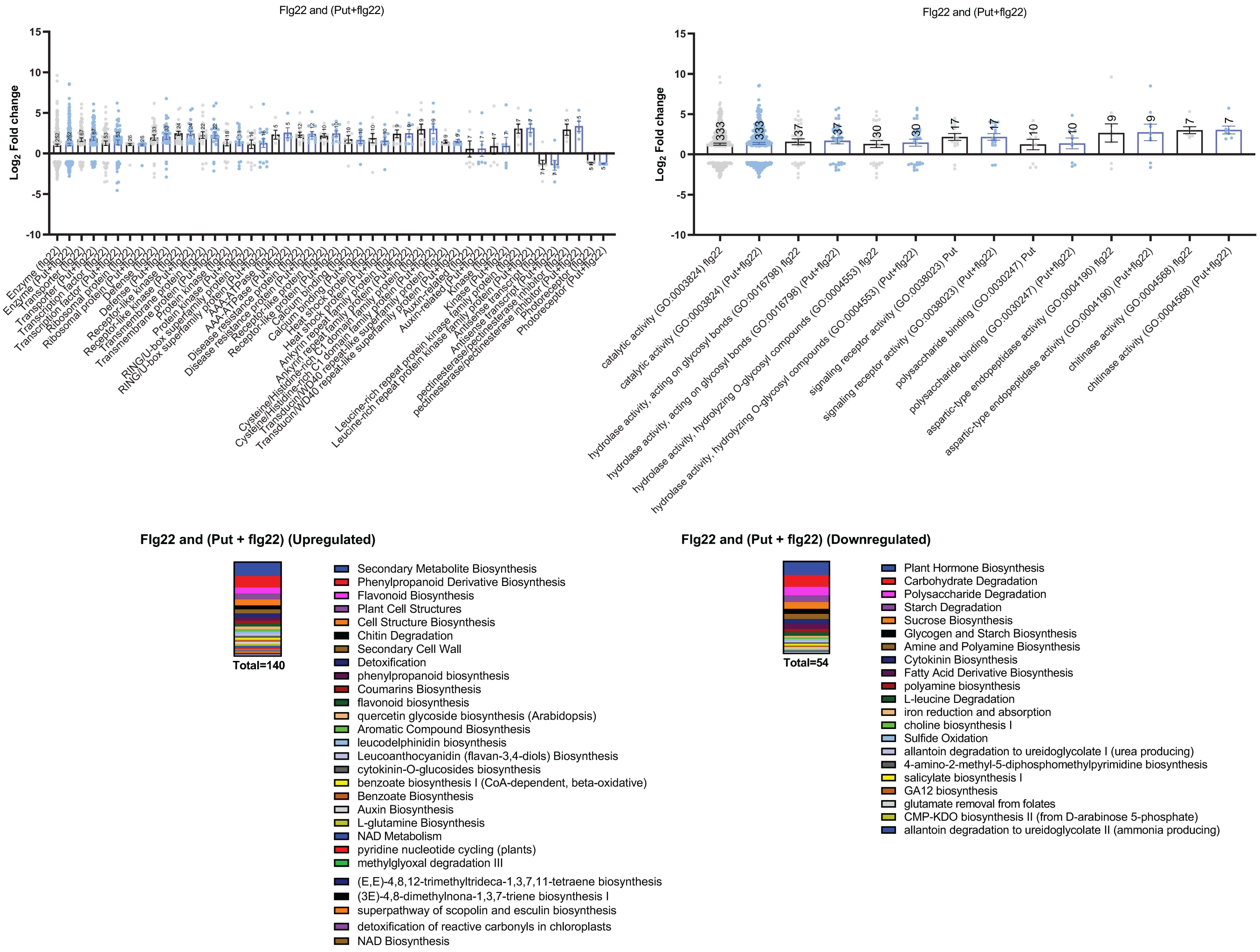

Figure S19. Molecular function categorization and metabolic pathway enrichment analysis of genes commonly deregulated in flg22 and (Put + flg22) treatments in the wild-type. Bars indicate the mean expression ± S.E in each category.

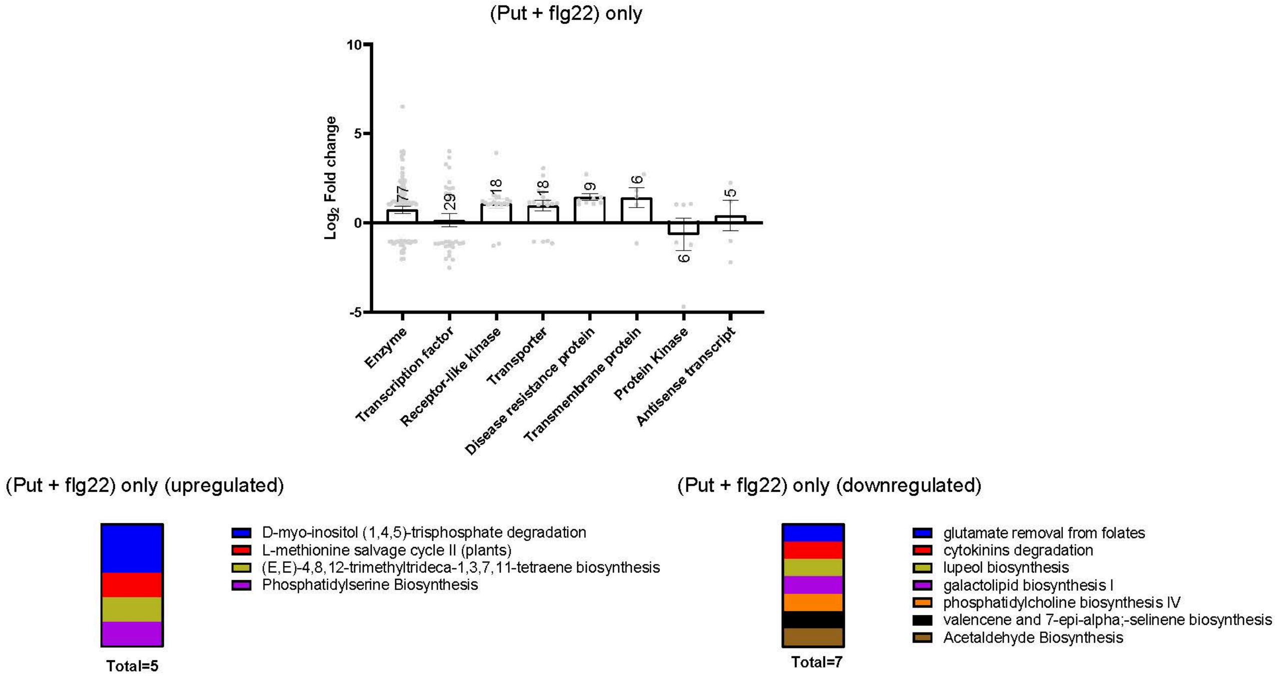

Figure S20. Molecular function categorization and metabolic pathway enrichment analysis of genes only deregulated in (Put + flg22) compared to flg22 and Put treatments. Bars indicate the mean expression ± S.E in each category.

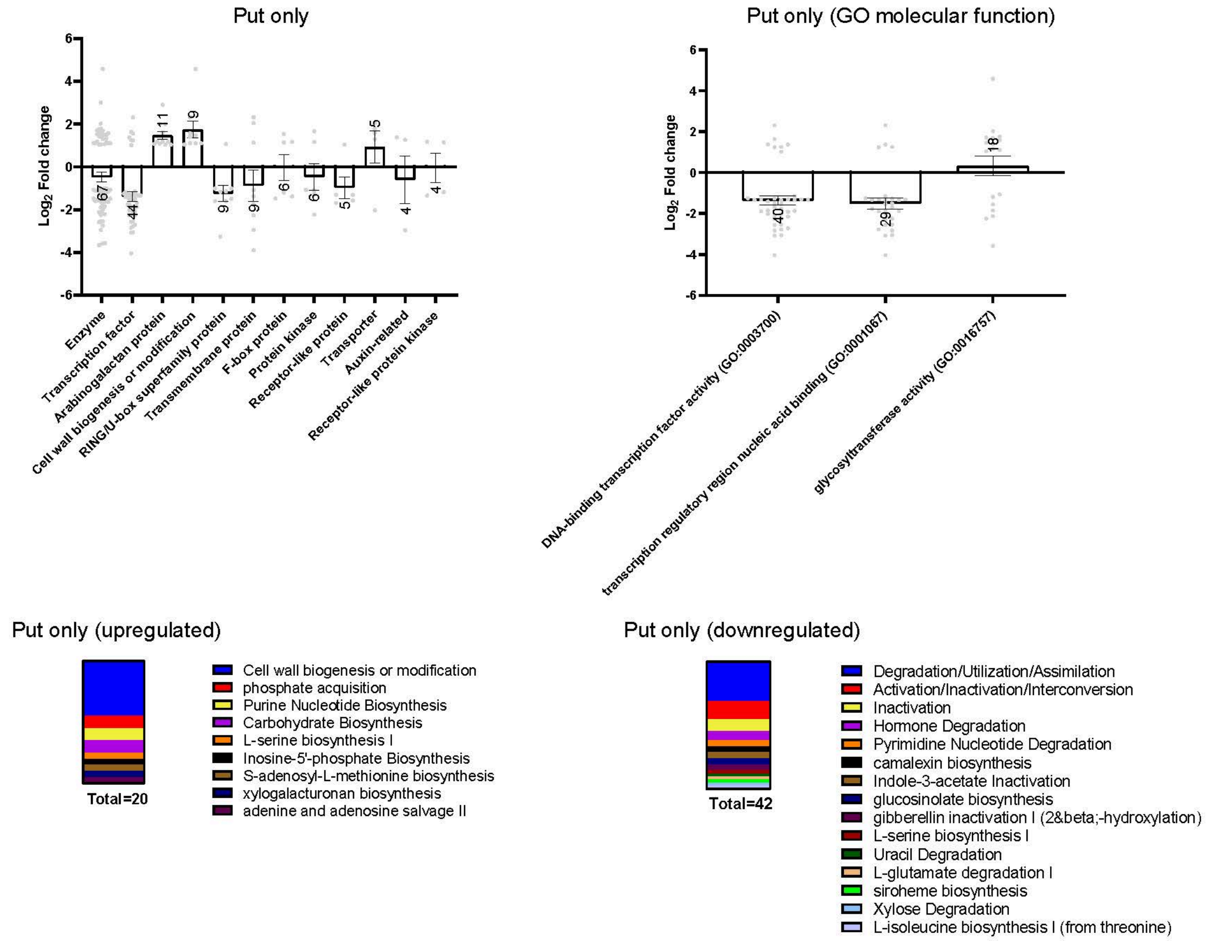

Figure S21. Molecular function categorization and metabolic pathway enrichment analysis of genes only deregulated by Put compared to flg22 and (Put + flg22) treatments. Bars indicate the mean expression ± S.E in each category.

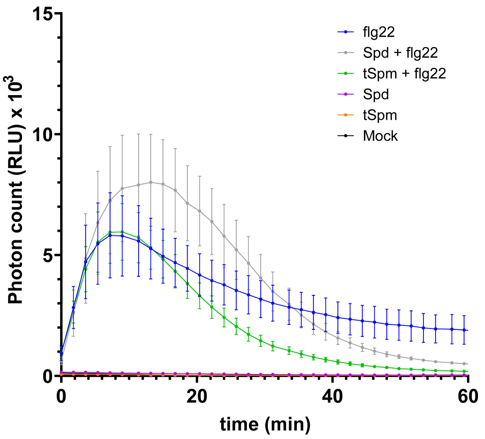

Figure S22. Effect of thermospermine (tSpm, 100 µM) and spermidine (Spd, 100 µM) on flg22-elicited ROS burst in the wild-type (Col-0). Leaf discs from 5-week-old plants were treated with flg22 (1 µM), tSpm (100 µM), Spd (100 µM), tSpm (100 µM) + flg22 (1 µM), Spd (100 µM) + flg22 (1 µM) or mock (water). Values represent the mean ± S.E. from at least twelve replicates per treatment and are expressed in photon counts (relative light units, RLU).
